# Skeletal High‐Strength Nanoporous Copper and Metamaterials: The Hakka Tulou Design Heritage

**DOI:** 10.1002/adma.202503701

**Published:** 2025-08-02

**Authors:** Haozhang Zhong, Tingting Song, Hongmei Liu, Chuanwei Li, Chenguang Li, Ming Wen, Zheda Ning, Jianfeng Gu, Ma Qian

**Affiliations:** ^1^ Institute of Materials Modification and Modeling Shanghai Jiao Tong University Shanghai 200240 China; ^2^ Centre for Additive Manufacturing School of Engineering RMIT University Melbourne VIC 3000 Australia; ^3^ School of Materials Science and Engineering Southwest Jiaotong University Chengdu 610031 China; ^4^ State Key Laboratory of Advanced Technologies for Comprehensive Utilization of Platinum Metals Kunming Institute of Precious Metals Kunming Yunnan 650106 China; ^5^ Shanghai Key Laboratory of Materials Laser Processing and Modification Shanghai Jiao Tong University Shanghai 200240 China

**Keywords:** additive manufacturing, copper, dealloying, metamaterials, nanoporous metals, segregation

## Abstract

Nanoporous metals (NPMs) are pivotal for next‐generation technologies, yet their inherent mechanical fragility has long hindered practical implementation. Drawing inspiration from the ingenious architecture of the ancient Hakka Tulou walls, a novel class of nanoporous copper (NPCu) materials—skeletal NPCu—is developed to overcome this limitation. To achieve this, we leverage the principles of solidification and dealloying in alloy design to engineer a unique two‐phase precursor alloy microstructure. This microstructure comprises a strong, ductile, non‐dealloyable skeletal phase (minor phase) and a readily dealloyable principal phase. Upon dealloying, the precursor transforms into skeletal NPCu, achieving exceptional yield strength (200.4 ± 15.2 MPa)—significantly surpassing that of conventional NPMs. Extending this design concept, we fabricate skeletal NPCu lattice metamaterials that surpass the Gibson‐Ashby strength model predictions by 800%, while offering an outstanding specific surface area (27.6 ± 1.2 m^2^ g^−1^) that enhances multifunctionality. By marrying ancient architectural wisdom with modern materials science, this innovation unlocks the vast potential of advanced NPMs for applications spanning energy, aerospace, and beyond.

## Graphical abstract

Inspired by the Hakka Tulou walls, skeletal nanoporous copper is fabricated via a two‐phase MnCu precursor and controlled dealloying. This strategy achieves high strength (200 MPa) and large specific surface area (27.6 m^2^/g), and extends to lattice metamaterials with hierarchical porosity, offering new design pathways for multifunctional metal architectures.

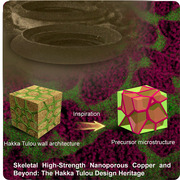

## 1 Introduction

Nanoporous metals (NPMs) are a unique class of metallic materials distinguished by their interconnected nanoscale pores, which endow them with an exceptionally high surface area. These materials have shown significant promise across a wide range of applications, including catalysis,^[^ ^1^ ^]^ energy storage,^[^ ^2^ ^]^ sensor technologies,^[^ ^3^ ^]^ and biomedicine.^[^ ^4^ ^]^ Since the introduction of dealloying in 1927 as an innovative manufacturing process to produce Raney nickel,^[^ ^5^ ^]^ it has remained the predominant method for fabricating large‐scale NPMs.

Despite their notable advantages, NPMs confront a critical and persistent challenge: their high porosity, while essential for their functionality, fundamentally undermines mechanical robustness, making NPMs vulnerable to structural instability or collapse under mechanical stress. Overcoming this trade‐off requires innovative strategies to fortify their mechanical strength without sacrificing their functional porosity—a crucial step toward ensuring reliable, stress‐resistant performance in real‐world applications.

Previous efforts to mitigate this challenge have explored structural and compositional modifications, such as

- Core‐shell architectures:^[^ ^6^ ^]^ Coating nanoporous aluminum with a native oxide shell improves load‐bearing capacity by supplementing structural reinforcement through the shell's rigidity.
- Ligament refinement:^[^ ^7^ ^]^ Reducing ligament sizes in NPMs leverages the size effect, where smaller dimensions inherently resist deformation, thereby enhancing strength.
- Hierarchical structuring:^[^ ^8^, ^9^ ^]^ Introducing nanoscale ligaments at sub‐architectural levels amplifies size‐dependent strengthening mechanisms, synergistically improving stiffness and fracture resistance.
- Nanovoid dispersion control:^[^ ^10^ ^]^ Optimizing the spatial distribution and minimizing the size of nanovoids strengthens dislocation‐void interactions, simultaneously boosting strength and ductility.

While these strategies have improved the mechanical properties and reliability of NPMs to some extent, the yield strength of NPMs remains comparatively low, typically below 100 MPa (see Table S1, Section S1, Supporting Information). This enduring limitation highlights the necessity for innovative design approaches to advance mechanical performance and meet the demands of high‐stress applications.

Among the various NPMs, nanoporous copper (NPCu) stands out as a particularly attractive candidate due to its exceptional intrinsic properties. These include high thermal and electrical conductivity,^[^ ^11^ ^]^ outstanding catalytic activity, especially in oxidation‐reduction reactions,^[^ ^12^ ^]^ and inherent antibacterial characteristics.^[^ ^13^ ^]^ Furthermore, NPCu offers a cost‐effective alternative to noble metals such as gold and platinum, broadening its appeal for diverse applications. However, akin to other NPMs, NPCu is constrained by its low strength and limited mechanical integrity (Table S1, Supporting Information), which impede its full utilization in advanced technologies.

In this study, through the design and fabrication of a new high‐strength NPCu, we introduce a novel class of NPMs—skeletal NPMs—engineered to dramatically improve mechanical strength and structural integrity. Below, we outline the conceptual framework and methodology employed to achieve this innovation. The design leverages fundamental principles of solidification and dealloying, offering a versatile and adaptable approach that could be extended to other alloy systems.

## 2 Conceptual Design

### 2.1 Architecting Dealloyed Microstructure: Introducing the Skeletal NPM Concept

**Figure 1:**
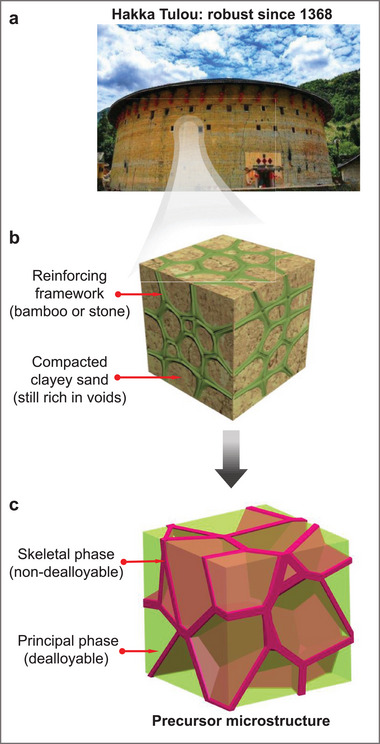
Skeletal NPCu mimicking the ingenious architecture of Hakka Tulou walls. a) A typical Hakka Tulou building, adapted from.^[^ ^16^ ^]^ b) Illustration of the architecture of Hakka Tulou walls, which consist of a bamboo framework embedded in compacted, yet void‐rich, earth. c) A precursor microstructure that mimics the Hakka Tulou wall architecture, designed through solidification‐driven solute micro‐segregation.

China's Hakka Tulou buildings^[^ ^14^ ^]^ represent a remarkable and enduring architectural achievement, exemplifying the ingenuity and resilience of traditional earthen construction. A notable example is Zhencheng Lou (**Figure** **1**a), which has maintained its structural integrity for over six centuries since its construction in 1368. These residential structures feature 1–2‐meter‐thick walls constructed with bamboo‐wood‐stone frameworks, embedded in compacted yet still void‐rich earth (Figure 1b). This ingenious design continues to influence modern earthen construction practices throughout Asia.^[^ ^15^ ^]^

Drawing inspiration from the centuries‐long mechanical resilience of Hakka Tulou walls, this study seeks to apply their design principles to develop innovative nanoporous materials (NPMs). Our concept involves constructing a 3D network of a strong, ductile, and non‐dealloyable skeletal phase within a readily dealloyable principal phase (Figure 1c), effectively compartmentalizing the nanoporous structure into discrete, self‐contained cells. This approach aims to localize ligament failure within individual cells, thus preserving the functional benefits of NPMs—such as high surface area—while addressing critical challenges like mechanical fragility and limited structural integrity. The following section further elaborates on our design strategies to realize this innovative concept.

### 2.2 Transforming Concept into Reality: Harnessing Alloy Solidification and Dealloying

To bring our design concept to life, the precursor alloy must possess two critical microstructural features: a strong, ductile, non‐dealloyable skeletal phase (minor phase) and a dealloyable principal phase (matrix phase). The skeletal phase provides structural integrity by maintaining the 3D framework after dealloying, while the principal phase enables the creation of nanoporous structures through controlled dealloying. Alternatively, the skeletal phase can be engineered to dealloy at a significantly slower rate than the principal phase, thereby ensuring that the structural framework remains intact after dealloying.

In order to identify suitable precursor alloys, we conducted a comprehensive analysis of Cu‐related binary phase diagrams.^[^ ^17^ ^]^ This analysis led us to identify Mn‐Cu alloys as ideal candidates for two key reasons: (i) they avoid the formation of brittle intermetallic phases, and (ii) they naturally segregate into Mn‐rich (dealloyable) and Cu‐rich (non‐dealloyable) phases during solidification. This unique behavior aligns perfectly with our design requirements and supports the development of the desired microstructure.

To further elucidate, consider the solidification process of a binary Mn‐X₀Cu alloy, where X₀ denotes the alloy composition in atomic percentage, with X₀ < 50 at.%. According to the Mn‐Cu equilibrium phase diagram,^[^ ^17^ ^]^ as solidification progresses, the solidified phase becomes enriched in Mn and depleted in Cu. This leads to an increasing Cu concentration in the remaining liquid, which becomes particularly pronounced when the liquid volume fraction (f_L_) drops below ≈20%. As a result, the as‐solidified microstructure consists of a Cu‐enriched skeletal phase (less than 20 vol.%) and a Cu‐depleted matrix phase (greater than 80 vol.%, denoted as f_S_). Using micro‐segregation models,^[^ ^18^, ^19^ ^]^ we can predict the average Cu and Mn compositions in both phases, accounting for solute back diffusion and the effect of cooling rate on the solute partition coefficient (*k*).

Notably, to preserve a non‐dealloyable Cu‐enriched skeletal phase alongside a readily dealloyable Cu‐depleted matrix, the respective copper contents X_L_(Cu) and X_S_(Cu) must meet critical thresholds—exceeding ≈50 at.% for the skeletal phase and remaining below roughly 40 at.% for the matrix—as governed by dealloying principles.^[^ ^8^, ^20^, ^21^ ^]^ Achieving this compositional balance requires an appropriately designed precursor alloy composition, tailored to the given solidification conditions (Figure S1, Supporting Information).

This study leverages laser‐based powder bed fusion (PBF‐LB), a leading additive manufacturing (AM) method, to fabricate Mn‐Cu precursor alloy and its lattice metamaterials. The cooling rate in PBF‐LB varies across a wide range, and we use an average value of 10⁵ K s^−1^ for designing our precursor alloys, since the cooling rate typically decreases with build height.^[^ ^22^ ^]^ Table 1 presents our predictions for the average Cu compositions in both the skeletal phase (remaining liquid) and the principal phase (solidified solid) for different Mn‐X₀Cu compositions (calculation details are provided in the Section S2, Supporting Information).

**Table 1:** Prediction of skeletal and principal phase compositions for different precursor Mn‐X_0_Cu alloys when solidified at the cooling rate of 10⁵ K s^−1^.

Precursor Mn‐X_0_Cu alloys	Skeletal phase (remaining liquid)		Principal phase (solidified phase)	
*X*_0_, at.%	*f*_L_, vol.%	*X*_L_(Cu), at.%	*f*_S_, vol.%	*X*_S_(Cu), at.%
44	20	60	80	40
40	20	55	80	37
**42**	**15**	**60**	**85**	**37**
38	15	55	85	34
39	10	60	90	34
35	10	55	90	33

Finally, to maintain a primarily nanoporous structure, we limit the non‐dealloyable skeletal phase to ≈15 vol.% (i.e., fs = 85 vol.%). Consequently, we select a Mn‐42 at.% Cu precursor alloy composition, highlighted in bold in Table 1. As will be demonstrated, the dealloying behavior of this Mn‐42 at.% Cu alloy aligns closely with the microsegregation‐based theoretical predictions in **Table** **1**. This alloy design, in the context of PBF‐LB, ensures the formation of a strong, ductile, non‐dealloyable skeletal network and a readily dealloyable matrix phase—essential for creating skeletal NPCu.

## 3 Results and Discussion

### 3.1 Microstructure of Skeletal NPCu

**Figure 2:**
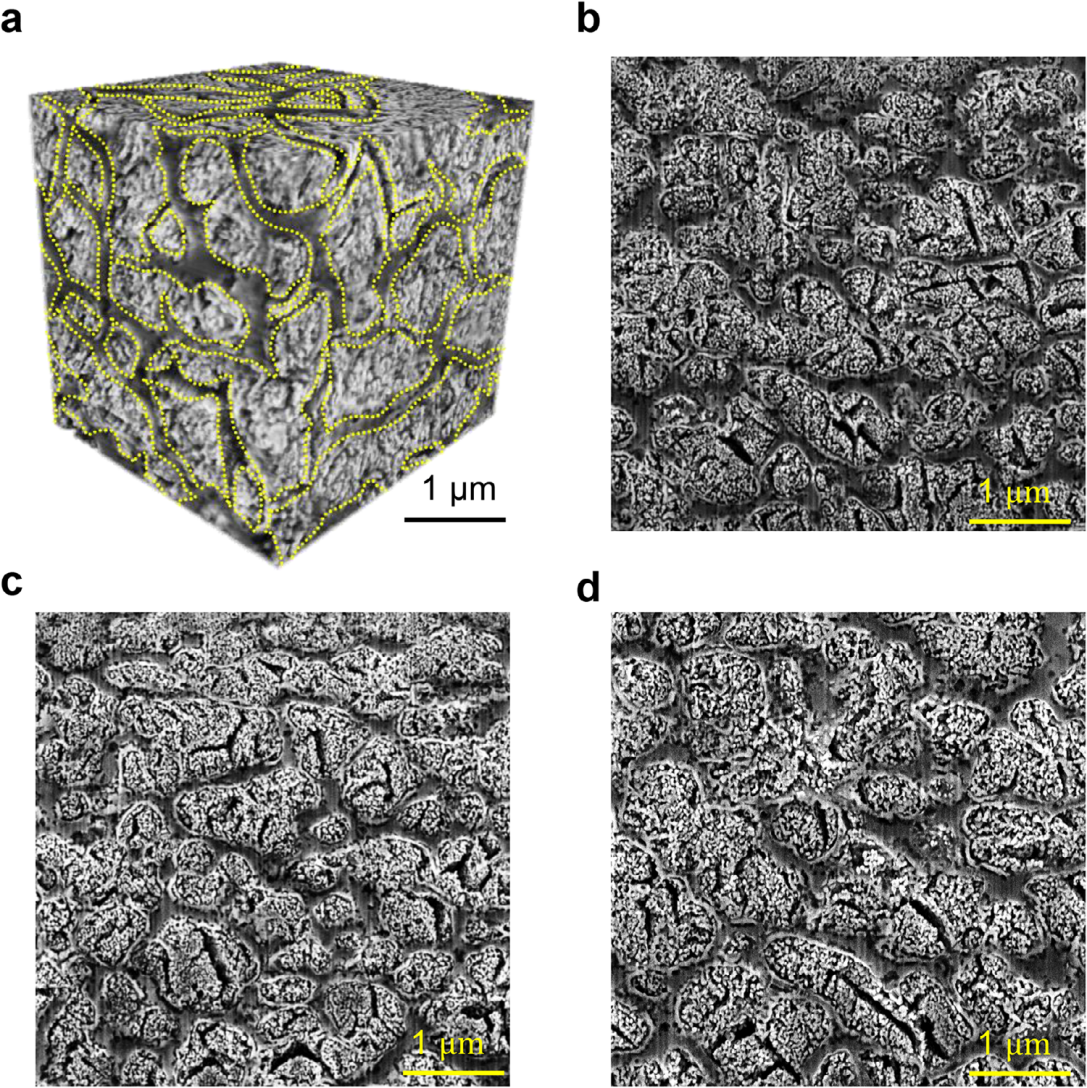
Dealloyed microstructure of the Mn‐42.0 at.% Cu alloy fabricated via PBF‐LB. a) A 3D reconstruction generated from 2500 FIB‐SEM images, captured within a 5 × 5 × 5 µm^3^ volume. b–d) Microstructures on the left (b), rear (c), and bottom (d) surfaces of the cuboid shown in (a).

The Mn‐42.0 at.% Cu precursor alloy was fabricated via PBF‐LB, and subsequently dealloyed in a 0.25 mol L^−1^ H_2_SO_4_ solution at 25 °C for 24 h. To reveal the three‐dimensional (3D) architecture of skeletal NPCu, a 3D reconstruction was carried out using 2500 focused ion beam‐scanning electron microscopy (FIB‐SEM) images, as shown in **Figure** **2**a. The yellow dashed contours outline the non‐dealloyable Cu‐enriched skeletal phase, revealing a three‐dimensionally interconnected skeleton at the microscale, as confirmed by the series of sectional analyses. Systematic views of the microstructure from various perspectives (Figure 2b–d) display distinct nanoporous Cu cells encapsulated by the skeletal phase. This unique microstructure, consisting of the non‐dealloyable skeleton and the nanoporous matrix, resembles the structural design of Hakka Tulou walls (Figure 1b).

To evaluate the dimensional stability of the precursor alloy during the dealloying process, we conducted comparative analyses on samples with varying dimensions (2  ×  2  ×  4 mm^3^ and 30  ×  30  ×  1 mm^3^). As shown in Figure S2 (Supporting Information), no measurable structural shrinkage was observed before and after dealloying. Moreover, the resulting microstructure exhibited no cracks or delamination between the nanoporous matrix and the Cu‐rich skeleton, indicating robust mechanical integrity throughout the microstructure under the dealloying conditions employed in this study.

Nano‐computed tomography data analysis (Figure 2) determined the volume fractions of the dark (Cu‐rich) and light (Mn‐rich) regions to be 19.3 ± 4.2% and 80.7 ± 5.1%, respectively. Additionally, transmission electron microscopy‐energy dispersive X‐ray spectroscopy (TEM‐EDS) mapping confirmed the precursor alloy composition to be 57.6 ± 3.4 at.% Mn and 42.4 ± 2.6 at.% Cu, matching our alloy design.

**Figure 3:**
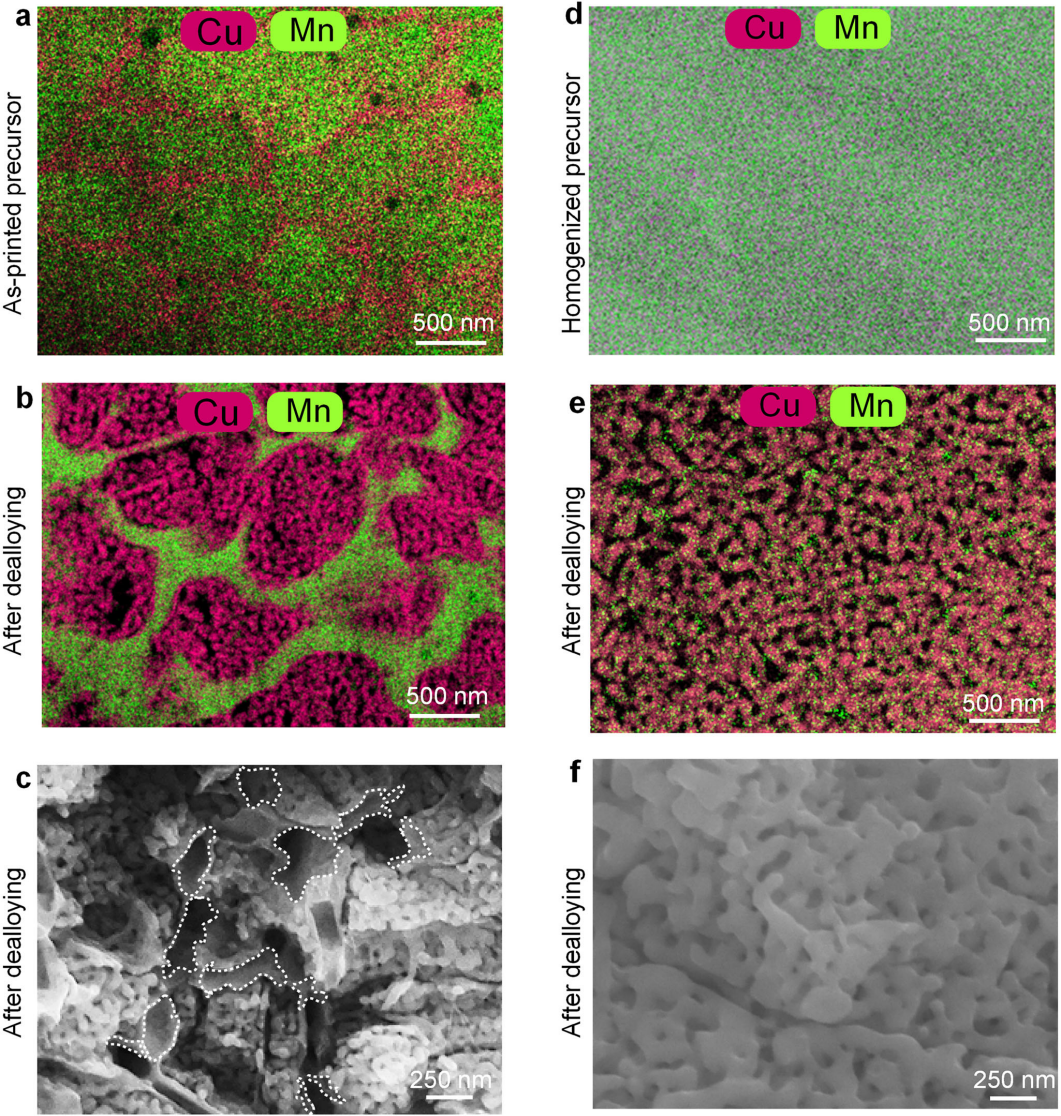
Microstructure of the Mn‐42 at.% Cu precursor alloy in various states. a) As‐printed (TEM‐EDS mapping), displaying the non‐dealloyable Cu‐rich skeleton (Mn‐52.4 ± 3.1 at.% Cu) and the dealloyable matrix (Mn‐31.8 ± 5.8 at.% Cu). b) Dealloyed, highlighting the retained non‐dealloyable skeletal phase (green) with a composition of Mn‐49.5 ± 1.1 at.% Cu, and the nanoporous matrix with a composition of Cu‐0.9 ± 0.1 at.% Mn. c) SEM image showing the skeleton, outlined by white dotted lines, and the ultrafine ligaments (42.5 ± 7.5 nm) within the nanoporous matrix. d) Homogenized (850 °C for 30 min), TEM‐EDS mapping exhibiting a nearly homogeneous microstructure. e) Skeleton‐free NPCu, dealloyed from the homogenized precursor in (d). f) SEM image revealing significantly coarser ligaments (77.4 ± 9.6 nm) in the skeleton‐free NPCu. Ligament size distributions are shown in Figure S9 (Supporting Information).

**Figure** **3**a–c examines the microstructure before (as‐printed) and after dealloying, revealing an ultrafine porous structure with elemental segregation in the precursor alloy. Notably, Cu enrichment occurs at cellular boundaries (52.4 ± 3.1 at.%), while Cu depletion is observed in intracellular regions (31.8 ± 5.8 at.%) (Figure 3a and TEM‐EDS line scan in Figure S3, Supporting Information). These observations align with the theoretical predictions presented in Table 1, confirming the expected behavior of the precursor alloy during dealloying.

Further analysis of the microstructure (Figure 3b) reveals two distinct phases: a non‐dealloyable skeletal phase (green) and a Cu‐depleted principal phase that undergoes dealloying to form NPCu (red). TEM‐EDS measurements indicate that the resulting bulk skeletal NPCu has a composition of 91.0 ± 1.3 at.% Cu and 9.0 ± 0.3 at.% Mn, with a relative density of ≈54% (see Ref. Note).^[^ ^23^ ^]^ However, the NPCu matrix itself contains only 0.9 ± 0.1 at.% Mn, with the elevated bulk Mn content primarily originating from the non‐dealloyable skeleton, which has a composition of Mn–49.5 ± 1.1 at.% Cu. The residual Mn in the NPCu matrix exists in three states—metallic (Mn⁰) and oxidized forms (Mn^2^⁺ and Mn^3^⁺), as shown in Figure S8 (Supporting Information). Based on the precursor alloy composition and TEM‐EDS analyses, the volume fraction of the skeletal phase was calculated to be 16.3 vol.%, the NPCu matrix accounting for 83.7 vol.% (Equation S14, Supporting Information). These values are consistent with theoretical estimates (f_L_ = 15%, Table 1) and show reasonable agreement with the CT‐estimated value of 19.3 vol.% (Figure 1), further validating the experimental results.

A critical finding is the role of the skeletal structure in stabilizing the NPCu structure. As shown in Figure 3c, the skeletal phase, represented by white dotted envelopes, acts as a geometric constraint, effectively mitigating ligament coarsening. This results in ultra‐fine ligaments with an average size of 42.5 ± 7.5 nm (Figure 3c, Figure S9a–c, Supporting Information). This secondary role of the skeleton highlights its importance in preserving the nanoscale integrity of the dealloyed structure. These SEM and TEM observations collectively confirm the formation of an intricate nanoporous structure, in which the skeleton serves to spatially confine and refine the nanoporous ligaments.

To further investigate the influence of the skeletal phase, skeleton‐free NPCu samples were fabricated for comparison. These samples were prepared by annealing the as‐printed Mn‐42.0 at.% Cu precursor alloy at 850 °C for 30 min to eliminate micro‐segregation before dealloying (Figure 3d and TEM‐EDS line scan in Figure S6, Supporting Information). The annealing temperature and duration were optimized through a series of trials at various temperatures (Section S3, Supporting Information), ensuring complete homogenization of the precursor alloy.

The resulting skeleton‐free NPCu microstructure, shown in Figure 3e,f, exhibits a homogeneous structure without the geometric constraints imposed by the skeletal phase. In this case, all Cu atoms contribute to the formation of the nanoporous structure, leading to significantly coarser ligaments. The average ligament size in the skeleton‐free NPCu (77.4 ± 9.6 nm, Figure 3f and Figure S9d–f, Supporting Information) is 82% larger than that observed in the skeletal NPCu (42.5 ± 7.5 nm, Figure 3c and Figure S9, Supporting Information). This stark contrast underscores the critical role of the skeletal phase in maintaining the ultrafine ligament morphology and highlights the trade‐offs between structural homogeneity and nanoscale feature preservation.

### 3.2 Mechanical Properties of Skeletal NPCu

**Figure 4:**
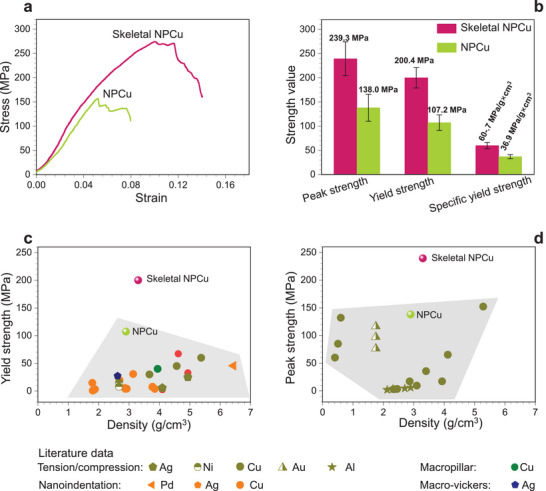
Mechanical properties of skeletal NPCu compared to existing NPMs. a) Compressive stress‐strain curves for skeletal NPCu (Cu‐9.0 at.% Mn) and skeleton‐free NPCu (Cu‐0.8 at.% Mn). b) Comparison of peak strength, yield strength, and specific yield strength between skeletal NPCu and skeleton‐free NPCu. See Table S3 for detailed comparisons. c‐d) Comparison of yield strength (c) and peak strength (d) between skeletal NPCu and existing NPMs (refer to Table S1 (Supporting Information) for detailed literature data).

Compression tests were conducted on skeletal NPCu and skeleton‐free NPCu samples with identical precursor alloy compositions. The results (**Figure** **4**a,b) demonstrate that skeletal NPCu exhibits a yield strength of 200.4 ± 21.1 MPa, which is 62% higher than that of skeleton‐free NPCu. Additionally, skeletal NPCu achieves a peak strength of 239.3 ± 35.0 MPa (70% higher than skeleton‐free NPCu) and a specific strength of 60.7 ± 6.5 MPa g^−1^ × cm^3^ (60% higher than skeleton‐free NPCu). These findings highlight the superior mechanical performance and structural efficiency of skeletal NPCu compared to its skeleton‐free counterpart.

Remarkably, both the absolute yield strength and specific yield strength of skeletal NPCu not only surpass those of previously reported NPMs (Figure 4c,d), but also exceed those of advanced structural variants, including hierarchical NPMs^[^ ^9^, ^24^ ^]^ and core–shell structured NPMs.^[^ ^6^ ^]^ Furthermore, its specific yield strength (60.7 MPa g^−1^) outperforms that of several state‐of‐the‐art strengthened Cu systems, such as micro‐ and nano‐SiC reinforced Cu (39.1 MPa cm^3^ g^−1^)^[^ ^25^ ^]^ or LaB₆ nanoparticle‐reinforced Cu (39.0 MPa cm^3^ g^−1^).^[^ ^26^ ^]^ Additionally, it is noteworthy that the yield strength of skeletal NPCu is comparable to that of widely used cast magnesium alloys—including Mg–9Al–1Zn, Mg–3Al–1Zn, and Mg–6Al–0.13Mn (all in wt.%)—which typically exhibit yield strengths ranging from 130 to 180 MPa.^[^ ^27^, ^28^, ^29^ ^]^ These alloys are widely regarded as representing the lower bound of strength for load‐bearing metals. This exceptional strength positions skeletal NPCu as a promising candidate for applications requiring lightweight, self‐supporting functional materials, particularly in engineering contexts where high strength and low weight are critical.

The enhanced mechanical properties of skeletal NPCu can be attributed to the synergistic contributions of both the skeletal phase and the NPCu matrix, with a detailed explanation provided in Sections S4 and S5, Supporting Information. The skeleton, composed of Mn‐49.5 at.% Cu, which is similar to the precursor alloy, has a yield strength that can be estimated based on the precursor alloy, as tested and shown in Figure S11b (Supporting Information). Given its volume fraction of 16.3%, the skeleton contributes ≈48.3 MPa to the overall strength (see Equations S13–S15, Supporting Information).

Meanwhile, the NPCu matrix contributes significantly due to its refined ligament structure. The yield strength of skeleton‐free NPCu, measured at 107.2 MPa (Figure 4b), serves as a baseline. By applying the Hall‐Petch relationship^[^ ^9^, ^30^ ^]^ and the bending‐dominated Gibson‐Ashby model,^[^ ^31^ ^]^ the refinement of ligaments from 77.4 to 42.5 nm increases the yield strength of the NPCu matrix from 101.2 MPa to 136 MPa (Equations S16 and S17, Supporting Information). This refinement contributes an additional 34.8 MPa.

Combining these contributions (48.3 MPa from the skeleton + 136.0 MPa from the NPCu matrix = 184.3 MPa) yields a theoretical strength that approaches the experimental value of 200.4 MPa. Notably, the skeleton accounts for 83.1 MPa of the total strength (48.3 MPa + 34.8 MPa = 83.1 MPa), representing 45% of the overall strength (83.1 MPa / 200.4 MPa = 45%). When compared to skeleton‐free NPCu, this contribution corresponds to 78% of its strength (83.1 MPa / 107.2 MPa = 78%), which generally agrees with the experimentally observed 62% enhancement in yield strength. This quantitative validation underscores the effectiveness of the Tulou‐inspired skeletal NPCu, which leverages geometric confinement and nanostructural control to achieve mechanical resilience far exceeding that of conventional NPCu.

As noted earlier, the nanoporous Cu matrix contains 0.9 ± 0.1 at.% residual Mn, present in both metallic and oxidized forms. Manganese oxides (e.g., MnO) are weak compounds, with a hardness of only ≈160 HV,^[^ ^32^ ^]^ compared to ≈1400 HV for alumina (Al_2_O_3_).^[^ ^33^ ^]^ Therefore, unlike Al_2_O_3_‐reinforced nanoporous aluminum,^[^ ^6^ ^]^ where the Al_2_O_3_ shell strengthens the ligaments to an estimated 1300 MPa and provides substantial load‐bearing support—the strengthening effect of these weaker manganese oxides is expected to be negligible. Moreover, due to the low residual Mn content in solid solution (0.9 at.%) and the nearly identical atomic radii of Mn (140 pm) and Cu (135 pm),^[^ ^34^ ^]^ the solid solution strengthening effect of Mn is also considered negligible.

### 3.3 Skeletal NPCu Strut Lattices: Conceptual Advancements Towards a New Frontier

Metallic lattices represent a cutting‐edge class of lightweight, multifunctional metamaterials that have gained widespread adoption across diverse industries,^[^ ^35^, ^36^, ^37^, ^38^ ^]^ driven by advancements in metal AM technologies. The integration of skeletal high‐strength NPCu struts into lattice designs offers a promising pathway to further enhance the functionality and performance of these metamaterials. Leveraging the breakthrough of skeletal high‐strength NPCu, we extend this design paradigm to engineer skeletal NPCu strut lattice metamaterials, pushing the performance of Cu‐based lattices to unprecedented levels.

**Figure 5:**
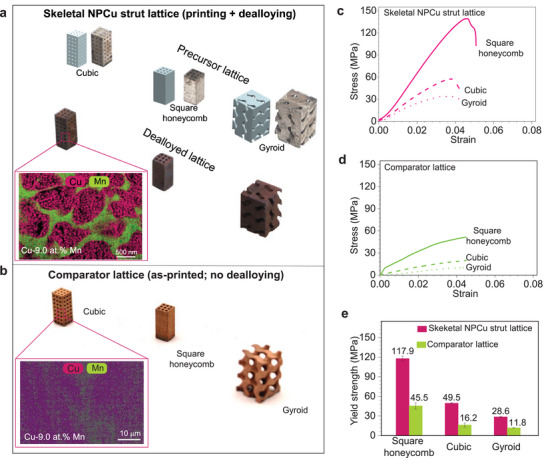
Mechanical property comparison of skeletal and comparator lattices with matched density (≈2.70 g cm^−3^) and composition (Cu‐9Mn). a) Computer‐aided design (CAD) and physical images of skeletal NPCu strut lattice precursors and dealloyed samples, including detailed views of ligament structures and compositional distribution. These structures were fabricated via a two‐step process combining PBF‐LB and Mn‐selective dealloying, enabling hierarchical micro‐nano porosity within each structure. b) Physical images of comparator lattices with corresponding strut compositional distribution (see Figure S10 for the microstructure of the as‐printed Cu‐9 at.% Mn, Supporting Information). c–d) Compressive stress–strain curves of skeletal NPCu and comparator lattices. e) Comparative analysis of yield strength between skeletal NPCu and comparator strut lattices.

To explore this concept, we designed three types of skeletal NPCu strut lattices: cubic, square honeycomb, and gyroid (**Figure** **5**a). These lattices were fabricated using a two‐step process combining PBF‐LB AM and Mn‐selective dealloying to achieve hierarchical micro‐nano porosity throughout each structure. First, precursor lattices with a nominal composition of Mn‐42.0 at.% Cu were fabricated via PBF‐LB AM. Subsequently, selective dealloying of these precursors yielded lattice struts with a final composition of 91 at.% Cu and 9 at.% Mn (Figure 5a). For comparison, we also fabricated comparator lattices with similar densities (≈ 2.7 g cm^−3^, Table S4, Supporting Information) and nearly identical compositions (91 at.% Cu and 9 at.% Mn) (Figure 5b). The relative densities of each lattice are detailed in Table S4 (Supporting Information).

Mechanical testing via uniaxial compression revealed striking differences in strength between the skeletal NPCu strut lattices (Figure 5c) and the comparator lattices (Figure 5d), as summarized in Figure 5e, despite both lattice types exhibiting similar deformation behavior. Notably, the skeletal NPCu strut lattices exhibit yield strengths (σ*) 2.4 – 3.1 times those of the comparator lattices, underscoring the substantial mechanical advantages conferred by the skeletal NPCu architecture. In particular, the high‐performance square honeycomb lattice, composed of the skeletal NPCu microstructure, surpasses the model by an impressive 800% and achieves a specific strength exceeding that of its fully dense Cu–9 at.% Mn base alloy. This exceptional strength is attributed to the inherent robustness of the Cu‐rich skeleton (Section 3.2), the refined ligament structure, and the hierarchical topology produced by the synergistic effects of PBF‐LB processing and dealloying (hierarchical strengthening).^[^ ^39^, ^40^ ^]^ Collectively, these co‐strengthening mechanisms effectively mitigate the typical trade‐off between porosity and mechanical performance in porous materials.

**Figure 6:**
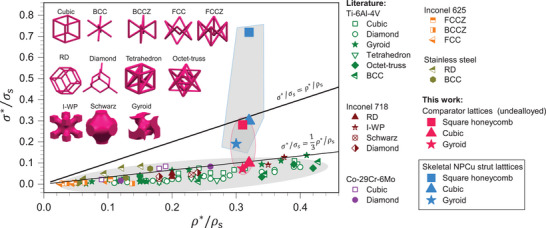
Mechanical properties comparison between skeletal NPCu strut lattices and existing lattices. RD: Rhombic dodecahedron, BCC: Body‐centered cubic. Data source: ref. [42].

This exceptional performance becomes even more evident when compared to classical scaling laws for porous metals. As illustrated in **Figure** **6**, most metallic lattices, including the comparator lattices, conform to or fall below the stretch‐dominated Gibson‐Ashby model.^[^ ^31^ ^]^ This model serves as a foundational empirical tool and general guideline for evaluating the mechanical behavior of lattice materials,^[^ ^41^ ^]^ and predicts σ*/σ_s_ = (ρ*/ρ_s_)/3, where ρ* and σ* are the density and yield strength of the lattice, respectively, and ρ_s_ and σ_s_ represent those of the base material. In contrast, our skeletal NPCu strut lattices defy this trend.

**Figure 7:**
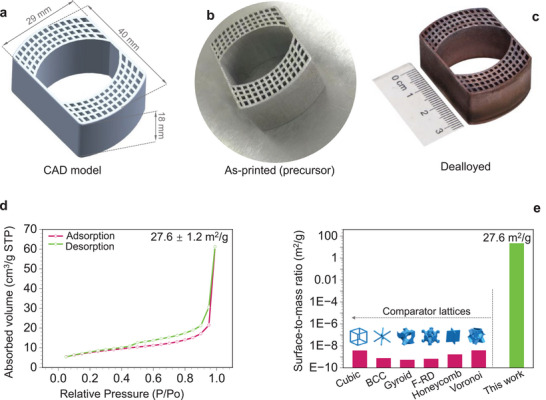
Conceptual advancements towards device development: A conformal skeletal NPCu strut square honeycomb lattice device. a–c) The device in its (a) designed, (b) as‐printed, and (c) dealloyed states. The as‐printed device has a composition of Mn‐42.0 at.% Cu, which transitions to Cu‐9 at.% Mn after dealloying. d) Brunauer–Emmett–Teller (BET) curves used to quantify the specific surface area. e) Comparison of the specific surface area between the micro‐lattice and the dealloyed sample, with the micro‐lattice specific surface area calculated using CAD models.

Integrating skeletal nanoporous structures into lattice designs thus not only expands the density‐strength design space for metallic materials but also overcomes the size limitations of traditional dealloying techniques. This approach enables hierarchical porosity with high surface area, allowing the creation of lightweight, ultra‐strong, and multifunctional standalone or conformal lattice devices that would be otherwise difficult to achieve. A notable example of this innovative approach is illustrated in **Figure** **7**, where we leveraged our experimental findings to design and fabricate a conformal skeletal NPCu strut square honeycomb lattice device, showcasing the potential of this technology.

As shown in Figure 7, the device was fabricated using a two‐step process: the initial design (Figure 7a) was printed via PBF‐LB, and subsequently dealloyed (Figure 7b), resulting in a conformal skeletal NPCu strut square honeycomb lattice structure with final dimensions of 40 × 29 × 18 mm^3^ (Figure 7c). This structure represents one of the largest dealloyed architectures reported to date, significantly surpassing the conventional dealloying limit, which is typically confined to thin films, surface layers, or bulk samples less than ≈2 mm thick.^[^ ^9^, ^24^ ^]^ This advancement opens the door to broader functional deployment of dealloyed architectures in practical engineering applications.

Further characterization of the device reveals a specific surface area of 27.6 m^2^ g^−1^ for the skeletal NPCu (Figure 7d), representing an improvement of ten orders of magnitude over conventional metallic lattices (Figure 7e) and approximately a threefold increase compared to most existing nanoporous metals (NPMs), which typically range from 10.77 to 12.27 m^2^ g^−1^.^[^ ^43^, ^44^ ^]^ This significant enhancement is attributed to the skeleton‐induced ligament refinement, as evidenced in Figures S9 and S13 (Supporting Information), with the lattice (dimensions: 40 × 29 × 18 mm^3^) containing ≈3 × 10^16^ ligaments. It offers a total surface area of 698 m^2^—equivalent to the surface area of three tennis courts—while maintaining a mass of only 25.3 g and occupying a volume of just 7.7 cm^3^. Notably, this stand‐alone conformal lattice device can sustain uniaxial compressive loads of up to 200 N mm^−^^2^ without permanent (plastic) deformation, highlighting its potential for functional applications under mechanical stress.

This work tackles a fundamental challenge in porous metal design and manufacturing: the simultaneous attainment of high strength and multifunctionality. By synergistically combining precursor alloy design, PBF‐LB AM and dealloying, we demonstrate that this goal is achievable. The approach hinges on using printable alloys that develop sufficient micro‐segregation during solidification, forming a robust, ductile, non‐dealloyable skeleton alongside a selectively dealloyable matrix—an architecture inspired by the structural wisdom of the Hakka Tulou walls.

Porous materials derive their functionality primarily from their extensive interfacial area, which facilitates enhanced surface reactions and interactions. The resulting structures are well‐suited for a broad range of multifunctional applications—including compact heat exchangers, catalytic reactors, antimicrobial surfaces, sensing devices, and drug delivery systems—while ensuring mechanical integrity in both space‐constrained conformal settings^[^ ^45^ ^]^ and stand‐alone engineering environments.

Looking ahead, fully realizing the potential of this approach will require overcoming several key challenges. These include the limited availability of alloys that are both printable and capable of undergoing micro‐segregation to enable targeted microstructural design for dealloying, the high cost associated with large‐scale production via PBF‐LB, and environmental concerns related to the use and handling of acid‐based dealloying processes.

## 4 Conclusion

In conclusion, our research has successfully tapped into the ancient architectural wisdom of Hakka Tulou walls, yielding a novel class of high‐strength NPCu, termed skeletal NPCu. By designing precursor Mn‐Cu alloys that harness solidification‐driven micro‐segregation, we engineered a unique two‐phase microstructure comprising a dealloyable Mn‐enriched primary phase and a non‐dealloyable, Cu‐enriched skeleton‐like secondary phase. Controlled dealloying of this precursor yielded skeletal NPCu with an exceptional yield strength of 200.4 ± 15.2 MPa and a remarkably high specific surface area of 27.6 m^2^ g^−1^. Building on this strategy, integration with laser‐based powder bed fusion additive manufacturing enabled the fabrication of ultra‐strong skeletal NPCu strut lattices with similarly outstanding specific surface areas and hierarchical porosity.

## 5 Experimental Section

### Metal Additive Manufacturing

*Powder Fabrication*: To achieve a printed Mn‐Cu precursor composition of Mn‐42 at.% Cu, we designed the nominal composition to be approximately Mn‐40at.% Cu (Table S6 and Section S6, Supporting Information). To account for Mn volatilization during PBF‐LB, the precursor powder was formulated with a composition of Mn: Cu = 60: 40 (at.%), targeting a final printed composition of Mn: Cu = 58: 42 (at.%) (Table S6, Supporting Information). This 2 at.% compensation for Mn loss was informed by prior literature^[^ ^46^ ^]^ and subsequently validated through TEM‐EDS analysis (Figure 3a). The powder was manufactured via gas atomization (Chengdu Huaying Powder Technology Co, Ltd). Figure S15 (Supporting Information) presented the powder morphology and size distribution, with D_50_ = 42.7 µm and D_90_ = 89.1 µm.

For Mn‐91.0 at.% Cu powders, used in the printing of comparator lattices, the fabrication process involved mixing irregularly shaped Mn powder (>99.89 wt.%), produced by electrolysis, with spherical Cu powder (>99.72 wt.%), obtained through gas atomization. To ensure uniform mixing of Mn and Cu powders, the powder blend was prepared using a resonant acoustic mixer (Shanghai Jinwan Co., Ltd.), followed by sieving to eliminate particles outside the target size range. The mixing protocol consisted of four consecutive cycles, each lasting 2 min at a vibration amplitude of 6 mm, with 1‐minute intervals between cycles. This sequence was repeated twice, with a 5‐minute intermediate cooling stage between the two sets. Finally, the powder for PBF‐LB was obtained after sieving through a 200‐mesh filter. The nominal compositions of the Mn‐42 at.% Cu and Mn‐91.0 at.% Cu powders used for printing are summarized in Table S6 (Supporting Information).

*Lattice Design*: The lattices were designed using SOLIDWORKS (Dassault Systèmes). Since the printed lattices were further lightened by dealloying, the initial designs featured a relative density of up to 80% to ensure the desired final density. The parts were then manually removed from the build plate using wire cutting. A 0.3 mm thick plate was added to the bottom of each lattice to compensate the material removal during wire cutting. The designed lattices are shown in Figure S16 (Supporting Information), and their topological parameters are listed in Table S5 (Supporting Information).

*PBF‐LB*: The process involves STL‐based design of the lattice structure, parameter selection for PBF‐LB, layer‐by‐layer printing of the Mn–Cu lattice, and subsequent powder removal. The TruPrinting 1000 system (TRUMPF Laser‐ und Systemtechnik GmbH) was employed to fabricate intricate Mn–Cu precursor lattices while simultaneously enabling the formation of the desired microstructure via micro‐segregation during solidification. The PBF‐LB parameters were listed in Table S7 (Supporting Information), with a substrate of stainless steel and a preheating temperature of 100 °C. After fabrication, the parts were cooled within the machine to room temperature (25 °C). The components were then manually removed from the build plate using wire cutting. Measured densities of printed alloys are summarized in Table S2 (Supporting Information). The printed skeletal NPCu strut lattice precursors and comparator lattices are shown in Figure S16 (Supporting Information). The precursor samples include sizes of 2  ×  2  ×  4 mm^3^ (for compression), 30  ×  30  ×  1 mm^3^ (for shrinkage evaluation), and lattice structures.

### Polymer Additive Manufacturing

The polymer lattices in Figure S14 (Supporting Information) were fabricated from commercially available polyacrylate‐based photosensitive resin (HTL resin, BMF Precision Technology Co., Ltd., China) via a microArch S240 projection microstereolithography system (BMF Precision Technology Co., Ltd., China). Their dimensional size was 9 × 9 × 9 mm^3^ and the relative densities were 35%.

### Thermal Treatment

The as‐printed Mn‐Cu samples were sealed in a quartz tube under an argon atmosphere, followed by heat treatment in a tube furnace at various temperatures and durations. The furnace (Nabertherm Heat Treatment Laboratory Furnaces) was preheated to the target temperatures of 450°C, 650°C, and 850°C. The samples were then placed in the furnace for the desired duration (30 min or 72 h). The precursor and dealloyed topological structures are presented in Figures S4–S7. To prepare comparison samples without non‐dealloyable skeletons, selected specimens were annealed at 850 °C for 30 min to homogenize composition and eliminate micro‐segregation (Figure 3f). Once the annealing time was complete, the samples were removed from the furnace and subjected to air cooling.

### Dealloying

The dealloying process was carried out in 0.25 mol L^−1^ H_2_SO_4_ solution (Kehua Co., Ltd) at 70°C to selectively remove Mn, thereby generating nanoporosity within the Mn‐enriched matrix phase while preserving the Cu‐rich skeleton (non‐dealloyable). The dealloying solution was refreshed approximately every 3 h throughout the process. After dealloying, the samples were carefully cleaned by rinsing and were stored in ethyl alcohol.

### The Material Characterization

*SEM*: The microstructure was characterized by SEM (TESCAN LYRA3, TESCAN ORSAY HOLDING, a.s.) using secondary electron mode at a voltage of 20.0 kV. Point EDS is applied to characterize the composition of Mn‐Cu powder.

*Electron Backscatter Diffraction (EBSD)*: EBSD analysis of the precursor alloy was conducted at an accelerating voltage of 20 kV and a tilt angle of 70°. The corresponding data was processed using Channel 5 software. Representative results are presented in Figures S3b, S4d, and S7d (Supporting Information).

*FIB*: FIB with a gallium (Ga) ion source was used to prepare TEM samples. The dual‐beam FIB system employed had a built‐in platinum (Pt) gas injection system, which deposited a thin layer of Pt on the region of interest. During operation, the ion beam current was applied to the selected region in a step‐down manner, with a final beam current of 100 pA used to minimize the impact of the ion beam on the area of interest.

*TEM*: TEM (JEOL 2100, 200 KV) was used to observe the dealloyed morphology at high magnification and analyze elemental distribution.

*The Atomic Force Microscope (AFM)*: AFM (MFP‐3D) was used to obtain nanomechanical information of the skeleton and NPM. The data were analyzed using AR Software version 13. The results are shown in Figure S12 (Supporting Information).

*3D FIB‐CT Reconstruction*: FIB‐CT provides 3D view information through reconstruction, enabling spatial visualization of the interpenetrating skeletal and nanoporous phases, which is essential for understanding their architecture and mechanical integrity. Three‐dimensional size of 5 × 5 × 5 µm^3^ was reconstructed. FIB scanning produced 2D images of 5 × 5 µm^2^ by Ga^+^ in the SEM system. And the space for each image is 20 nm, with a spacing of 20 nm between each image. After obtaining multiple two‐dimensional images, Dragonfly was used for the 3D reconstruction.

*Brunauer–Emmett–Teller (BET)*: Specific surface area is a fundamental property of nanoporous materials and is critical for a wide range of functional applications. In this study, the specific surface area of the dealloyed samples was measured using the standard BET method with a Quadrasorb Evo analyzer (Quantachrome Instruments, USA), following established procedures.^[^ ^47^ ^]^ Three 0.5 mm thick as‐dealloyed disks were analysed, which weighed 0.36 g, 0.31 g, and 0.34 g, respectively. All samples were degassed at 373 K for 16 h. The analysis bath temperature (liquid nitrogen) was 77.3 K. The equilibration time was 15 s.

*X‐Ray Diffraction (XRD)*: XRD was conducted on the Mn–42.0 at.% Cu alloy powder using a Bruker D8 Advance Diffractometer at 40 kV with a Cu Kα radiation source (wavelength). The scanning speed was 5° min^−1^. The result is presented in Figure S15d (Supporting Information).

*Powder Size Distribution*: Laser particle size analyzer (S3500) was used to analyze the powder size distribution of the Mn–42.0 at.% Cu alloy powder. The results are presented in Figure S15a–c (Supporting Information).

*X‐Ray Photoelectron Spectroscopy (XPS) Analysis*: Depth profiling was conducted on rod‐shaped skeletal NPCu specimens (0.5 mm in height, 4 mm in diameter) using a Thermo Fisher Scientific ESCALAB QXi XPS system. The ion gun operated in monatomic mode with an ion energy of 1000 eV. A laser spot diameter of 500 µm and a raster size of 2.70 mm were used—sufficient to capture representative information from the sample. The estimated sputtering rate was 0.05 nm/s, based on calibration with Ta_2_O_5_.

### The Mechanical Test

*Pillar Compression*: To evaluate and understand the intrinsic strength of individual struts in skeletal NPCu lattices, pillar compression tests were conducted on 7 µm‐diameter nanoporous micropillars, complementing bulk compression tests. Two pillars were characterized using a FIB system. Nanoindentation was performed with a Hysitron TriboIndenter equipped with a diamond flat‐ended tip (20 µm diameter) in displacement‐controlled mode. Pillar #1 had a height of 9.3 µm, a bottom diameter (d₁) of 5.9 µm, and a top diameter (d_2_) of 9.0 µm. Pillar #2 had a height of 9.6 µm, a bottom diameter of 6.8 µm, and a top diameter of 5.6 µm. The equivalent diameter of each pillar was calculated as (d₁ + d_2_)/2. Pillar compression tests reveal a yield strength of 222.4 MPa for skeletal NPCu (Figure S11, Supporting Information), closely matching the macroscopic value of 200.4 MPa and reaffirming its high strength.

*Macro‐Sample Compression*: The MTEST5000W Tensile/Compression Stage (GATAN, United Kingdom) was applied with a customized fixture. The dealloyed cuboid sample size was 2 × 2 × 4 mm^3^ (Figure S17c, Supporting Information), with a compression rate of 0.1 mm s^−1^.

*Lattice Compression*: These lattice samples were mechanically tested in a calibrated 50 kN Instron machine at a speed of 1 mm min^−1^ according to the ISO 13314 standard for ductility testing of porous materials.^[^ ^48^ ^]^ For porous and cellular metals, the yield stress is determined using the 0.2% offset method. This method involves drawing a line, known as the quasi‐elastic gradient line, parallel to the initial elastic portion of the stress‐strain curve. A straight line offset by 0.2% strain along the horizontal axis and parallel to the quasi‐elastic gradient line is then drawn. The yield stress is identified as the point where this offset line intersects the stress‐strain curve.

## Conflict of Interest

The authors declare no conflict of interest.

## Author Contributions

Conceptualization: Haozhang Zhong, Jianfeng Gu, and Ma Qian, Methodology: Haozhang Zhong, Tingting Song, Hongmei Liu, Ming Wen, Zheda Ning, Chuanwei Li, Chenguang Li, and Zheda Ning, Investigation: Haozhang Zhong, Tingting Song, Jianfeng Gu, and Ma Qian, Project Administration: Haozhang Zhong, Jianfeng Gu, and Ma Qian, Supervision: Jianfeng Gu and Ma Qian, Critical Analysis: Haozhang Zhong and Ma Qian, Writing: Haozhang Zhong and Ma Qian, Writing (review): All authors.

## Supporting information



Supporting Information

## Data Availability

The data that support the findings of this study are available from the corresponding author upon reasonable request.
