# Supplementary material for: Skeletal High‐Strength Nanoporous Copper and Metamaterials: The Hakka Tulou Design Heritage
Source: Adv Mater. 2025 Aug 2;37(47):e03701. doi: 10.1002/adma.202503701 (PMC12651096; doi:10.1002/adma.202503701)
Supplement: Supplementary file 1 — Supporting Information [file ADMA-37-e03701-s001.pdf]

ADVANCED MATERIALS

Supporting Information

for *Adv. Mater.*, DOI 10.1002/adma.202503701

Skeletal High-Strength Nanoporous Copper and Metamaterials: The Hakka Tulou Design Heritage

Haozhang Zhong, Tingting Song, Hongmei Liu, Chuanwei Li, Chenguang Li, Ming Wen, Zheda Ning, Jianfeng Gu and Ma Qian**

Supporting Information

1. The reported mechanical properties of nanoporous materials

Table S1 shows that nanoporous metals produced through dealloying exhibit relatively poor mechanical properties, with yield strengths on the order of 100 MPa.

Table S1 The reported mechanical properties of nanoporous metals.

NPM	Relative density	Density (g/cm^3)	Yield strength (MPa)	The first maximum strength (MPa)	Test method	Literature
NP-Cu	0.2015	3.769472	0.9		Compression	[1]
	0.32	1.835008	5.5			
	0.4207	2.877056	7.2			
	0.2048	3.773952	1.5			
	0.3211	1.880704	4			
	0.4212	2.926336	7.7			
	0.2099	3.846528	3.2			
	0.3266	3.769472	4.2			
	0.4293	1.835008	3.9			
NP-Cu	0.439	3.93344		17.2	Compression	[2]
	0.346	3.10016		9.5		
	0.274	2.45504		3.4		
	0.261	2.33856		2.9		
NP-Cu	0.32	2.8672		17.2	Compression	[3]
	0.38	3.4048		35.6		
	0.46	4.1216		65.2		
	0.59	5.2864		152.2		
NP-Cu	0.6	5.376	60	132	Compression	[4]
	0.51	4.5696	45	85		
	0.41	3.6736	30	60		
NP-Cu	0.44	3.9424	40		Macropillar	[5]
NP-Cu	0.2	1.792	14.7		Nanoindentation	[6]
	0.3	2.688	19.7			
	0.35	3.136	30.7			
NP-Au	~0.11-0.24	1.7-6.0	~ 1-20		Compression	[7]
NP-Ag	0.44	4.62	67.2		Nanoindentation	[8]
	0.47	4.935	32.7			
	0.39	4.095	3.71			
	0.39	4.095	2.56			
	0.47	4.935	24.7		Tension	[8]
	0.39	4.095	5.75			
	0.39	4.095	5.27			
NP-Ag	0.25	2.625	70		Nanoindentation	[6]
	0.25	2.625	27.3		Micro-Vickers	[6]
NP-Al	0.65	1.755		76.4	Tension	[9]
	0.65	1.755		97.4		
	0.65	1.755		116.4		
NP-Au	0.36	6.9552	100		Micropillar	[10]
NP-Au	0.25	4.83	70		Micropillar	[11]
NP-Pd	0.3	6.435	45.7		Nanoindentation	[6]
NP-Ni	0.3	2.6706	15.1		Tension	[12]
	0.3	2.6706	7.8			

2. The micro-segregation analysis

Fig. S1 schematically illustrates that elemental segregation across the boundary of dealloying threshold, is required to achieve our design concept mimicking the Hakka Tulou architecture. This process allows for partial dealloying, where the sacrificial element-enriched region undergoes

dealloying (green region in **Fig. S1**), while the noble element-enriched region remains intact (red region in **Fig. S1**) , forming the desired skeletal structure.

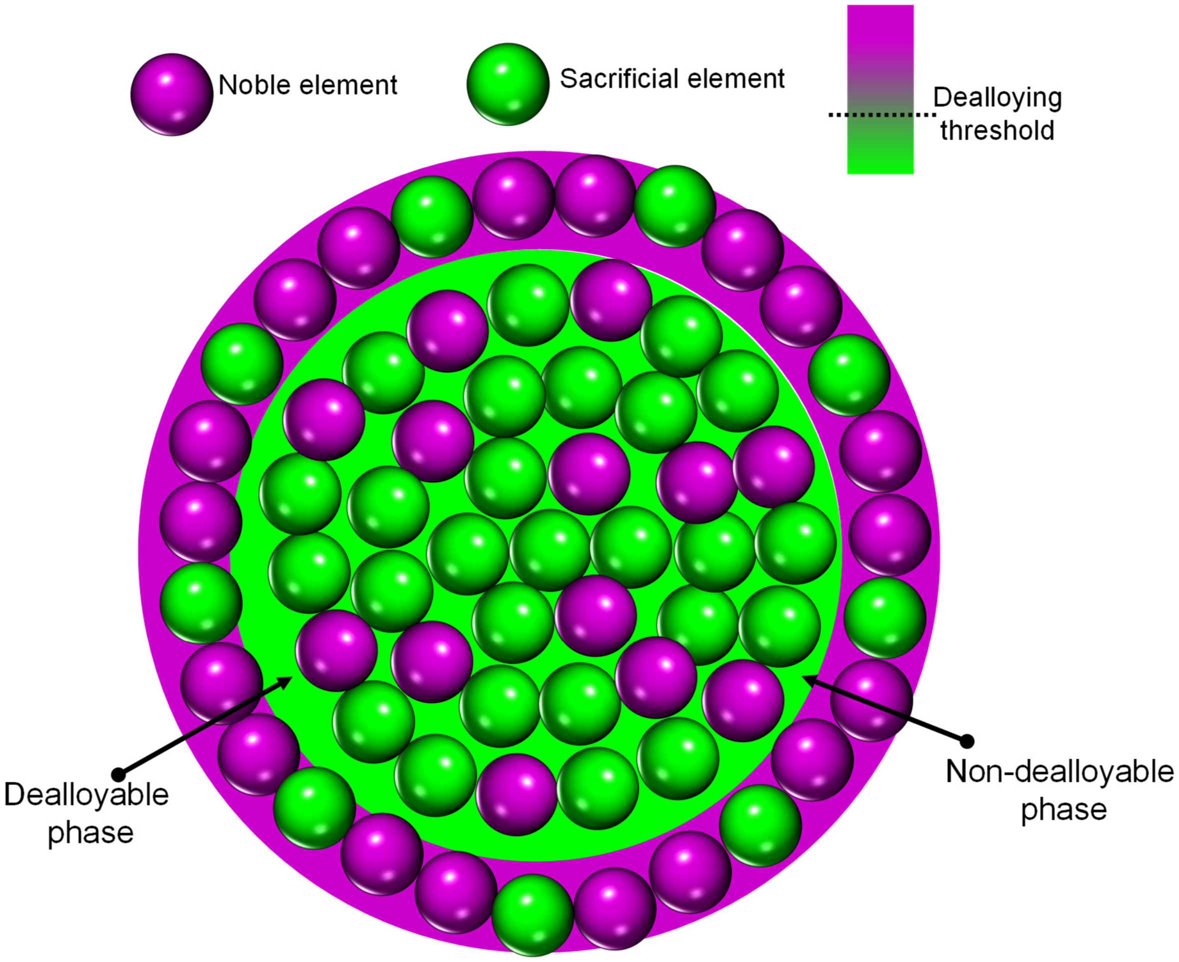

Fig. S1 Schematic illustration showing the elemental segregation related to the skeletal NPCu.

The Scheil equation is a well-regarded microsegregation model [13, 14] for predicting the Mn-Cu alloy [13, 14], such as Mn-Cu, when the solid phase volume fraction is between 80-90% [13, 15] and the solid diffusion coefficient of Cu, D_s (Cu), less than 10^{-12} m^2/s [16].

$$X_L(Cu) = X_0(\mathrm{Cu})(1 - f_s)^{k-1} \quad \text{S1}$$

where X_L(Cu) is Cu content in remaining liquid composition, X_0 is Cu content in alloy, f_s is solid phase volume fraction. k is the partition coefficient.

It is well known that rapid solidification causes a significant deviation from local equilibrium at the solid-liquid interface [17-19]. At a cooling rate of approximately 10^4 K/s, the transition from local equilibrium to non-equilibrium starts [17]. These deviations from chemical equilibrium at the solid-liquid interface are characterized by the velocity-dependent partition coefficient, k_v [18, 19], which can be expressed as shown in **Eq. S2**.

$$k_v = \frac{k_e + \frac{v}{v_D}}{1 + \frac{v}{v_D}} \quad \textbf{S2}$$

where v is the solid-liquid interface growth velocity, and $v_D = D_L/a_0$ represents the atomic diffusion speed at the interface, with a_0 being the interatomic spacing. For cooling rates in the range of 10^5-10^7 K/s [20](PBF-LB, due to the presence of thermal cycles, experiences significant variations in cooling rates, but generally remains within this range), the corresponding v ranges from 10 m/s to 1000 m/s [21]. D_L is the liquid diffusion coefficient of Cu in Mn, which is approximately $7.5 \times 10^{-9} \mathrm{m}^2/\mathrm{s}$ [22], and $a_0 = 2.66 \times 10^{-10} \mathrm{m}$ [23].

At the late solidification stage, the remaining liquid phase ultimately evolves into a Cu-rich phase. This Cu-rich phase eventually solidifies into what is referred to as a non-dealloyable phase, forming the reinforcing skeletons after dealloying. To prevent dealloying in this phase, it needs to satisfy:

$$X_L(\mathrm{Cu}) > 50 \text{ at.\%} \quad \textbf{S3}$$

Conversely, the existing solid phase corresponds to a dealloyable phase, forming nanoporous matrix after dealloying. To ensure that dealloying occurs effectively in this phase, it needs to satisfy:

$$X_S(\mathrm{Cu}) < 40 \text{ at.\%} \quad \textbf{S4}$$

For Mn- X_0Cu alloys with k <1 (positive segregation, k is the partition coefficient, see S-Eq. 1), significant solute enrichment begins to appearat the late stage of solidification. In such stage:

The volume fraction of Cu-rich phase (or reinforcing skeleton) in the alloy, represented by $(1 - f_s)$, should be carefully maintained to ensure that the Cu-rich phase contributes sufficient strength while preserving the nanoporous features. Besides, for Mn- X_0Cu alloys with k <1 (positive segregation), significant solute enrichment begins to appearat the late stage of solidification where $f_s > 80\%$. Under such dual constrait, we suggest keeping this fraction within these specified limits:

$$10\% \leq 1 - f_s \leq 20\% \quad \textbf{S5}$$

$X_s(\mathrm{Cu}) = k_v X_L(\mathrm{Cu})$ calculateds the solute concentration in solid at the liquid-solid interface [24, 25] rather than throughout the entire solid. Instead, we apply mass conservation to determine $X_S(\mathrm{Cu})$, as follows:

$$X_s(\mathrm{Cu}) = [X_0(\mathrm{Cu}) - X_L(\mathrm{Cu})(1 - f_s)]/f_s \quad \textbf{S6}$$

Utilizing **Eq. S1** and incorporating the constraints from **Eqs. S2-S6**, the corresponding compositions

(at.%) of $X_L(\mathrm{Mn}), X_S(\mathrm{Mn}), X_0(\mathrm{Cu})$ at different f_s ranging from 10% to 20% (**Eq. S5**) and at different cooling rates ranging from 10^5 to10^7 K/s, are calculated and given in ***Table S2***.

To illustrate the calculation, consider an example where $f_s = 85$, $X_L(Cu)$=60.0at.%, $k_v = 0.81$, (calculated using **Eq. S1** with a cooling rate of 10^5 K/s). Substituting these values into **Eq. S1** yields $X_0(Cu) = 42.0$ at.% and $X_S(Cu) = 37.0$ at.%, as summarized in **Table 1**.

3. Characterizations of precursors and dealloyed topologies

To assess dimensional stability, precursor alloy samples of different sizes ($2 \times 2 \times 4\ mm^3$ and $30 \times 30 \times 1\ mm^3$) were examined before and after dealloying. As shown in **Fig. S2**, no detectable shrinkage occurred, indicating high geometric fidelity. Moreover, the architecture remained free of cracks or interfacial separation between the nanoporous matrix and Cu-rich skeleton, reflecting strong mechanical and interfacial robustness under the processing conditions.

Fig. S3 presents the characterization of the dealloyed topologies alongside the precursor in its as-printed state. To facilitate a comparative study between skeletal-retained and skeletal-free dealloyed topologies, thermal treatment was applied to eliminate elemental segregation, the primary factor driving skeleton formation.

Various time-temperature combinations were explored, including heat treatments at 450°C for 30 minutes (**Fig. S4**) and 650°C for 30 minutes (**Fig. S5**), which did not fully eliminate segregation and may have impacted the formation of the skeleton in subsequent steps. In contrast, the heat treatment at 850°C for 30 minutes (**Fig. S6**) effectively removes the segregation while minimizing excessive Mn volatilization. However, when the treatment time was extended to 850°C for 72 hours (**Fig. S7**), significant Mn volatilization was observed, which affected the overall dealloying process. Therefore, 850°C for 30 minutes is identified as the optimal condition for obtaining the skeleton-excluded dealloyed topology.

Fig. S9 shows the ligament length for both skeletal-retained and skeletal-free dealloyed topologies, supplementary to the ligament diameter data presented in the main text. **Fig. S10** presents the microstructures of Mn-91.0at.%Cu, which corresponds to the final composition of skeletal NPCu. The alloy was printed, and subsequent mechanical tests were conducted to investigate the relative strength of the skeletal NPCu strut lattice.

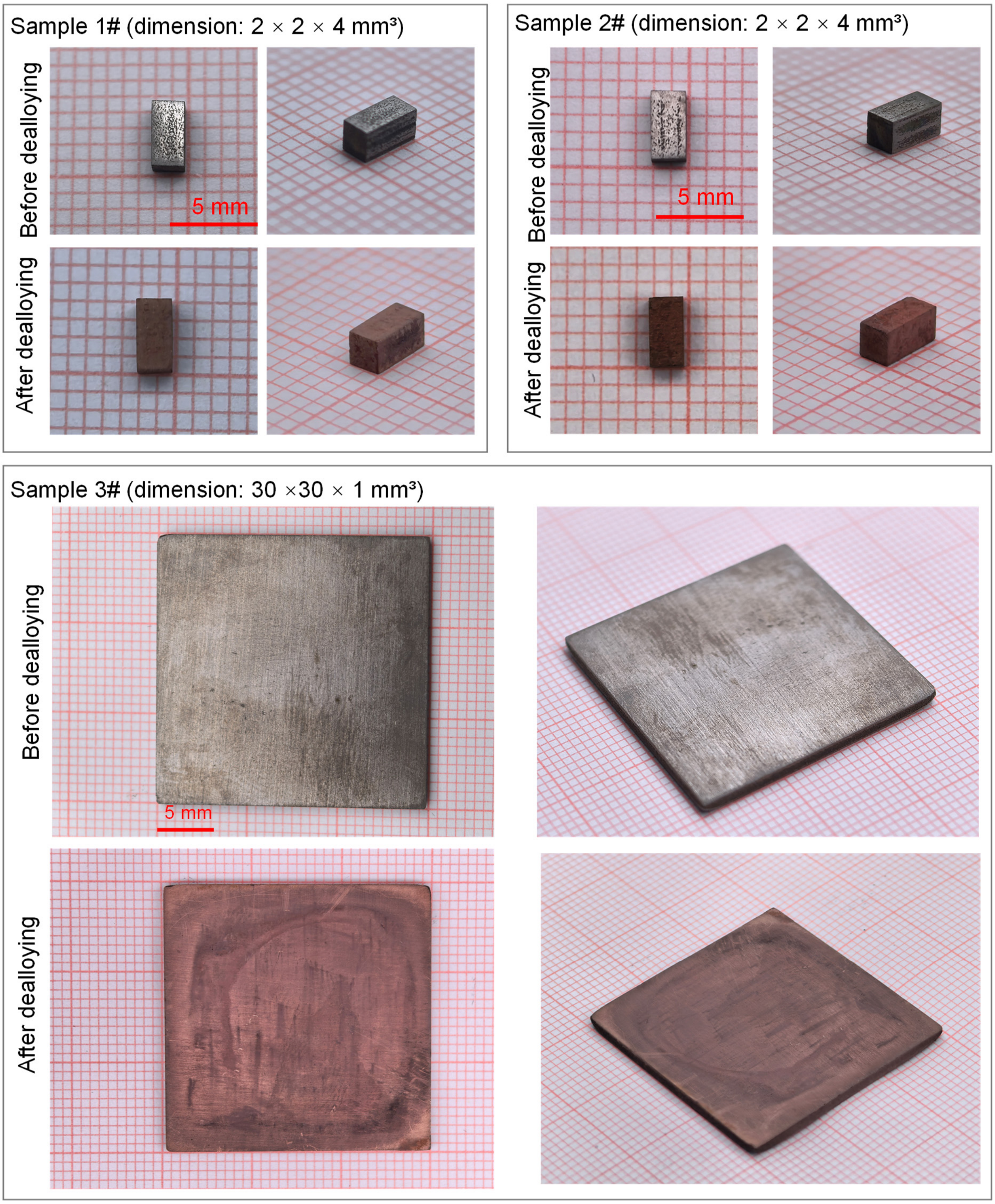

Fig. S2 Macroscopic comparison of skeletal NPCu precursor alloy (Mn-42 at.% Cu) samples before and after dealloying. Samples across scales (2×2×4 mm³ to 30×30×1 mm³) maintain dimensional stability after dealloying.

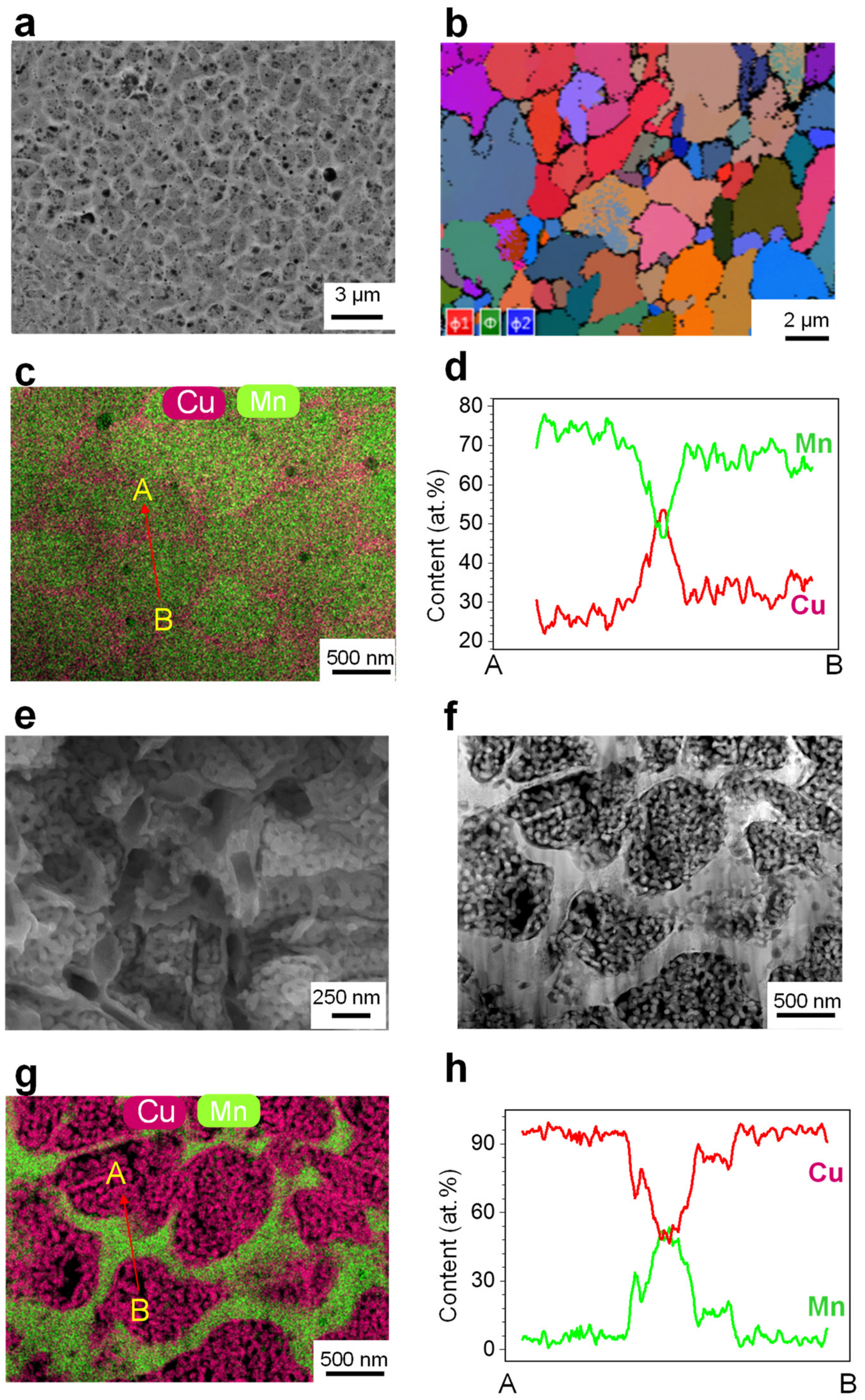

Fig. S3 **Characterization of dealloyed topologies and the PBF-LB precursor.** (a-d) Microstructures and compositions of precursors of Mn-42.0at.%Cu. (e-h) Dealloyed topologies and corresponding compositions. *e and g are recycled from the main text.*

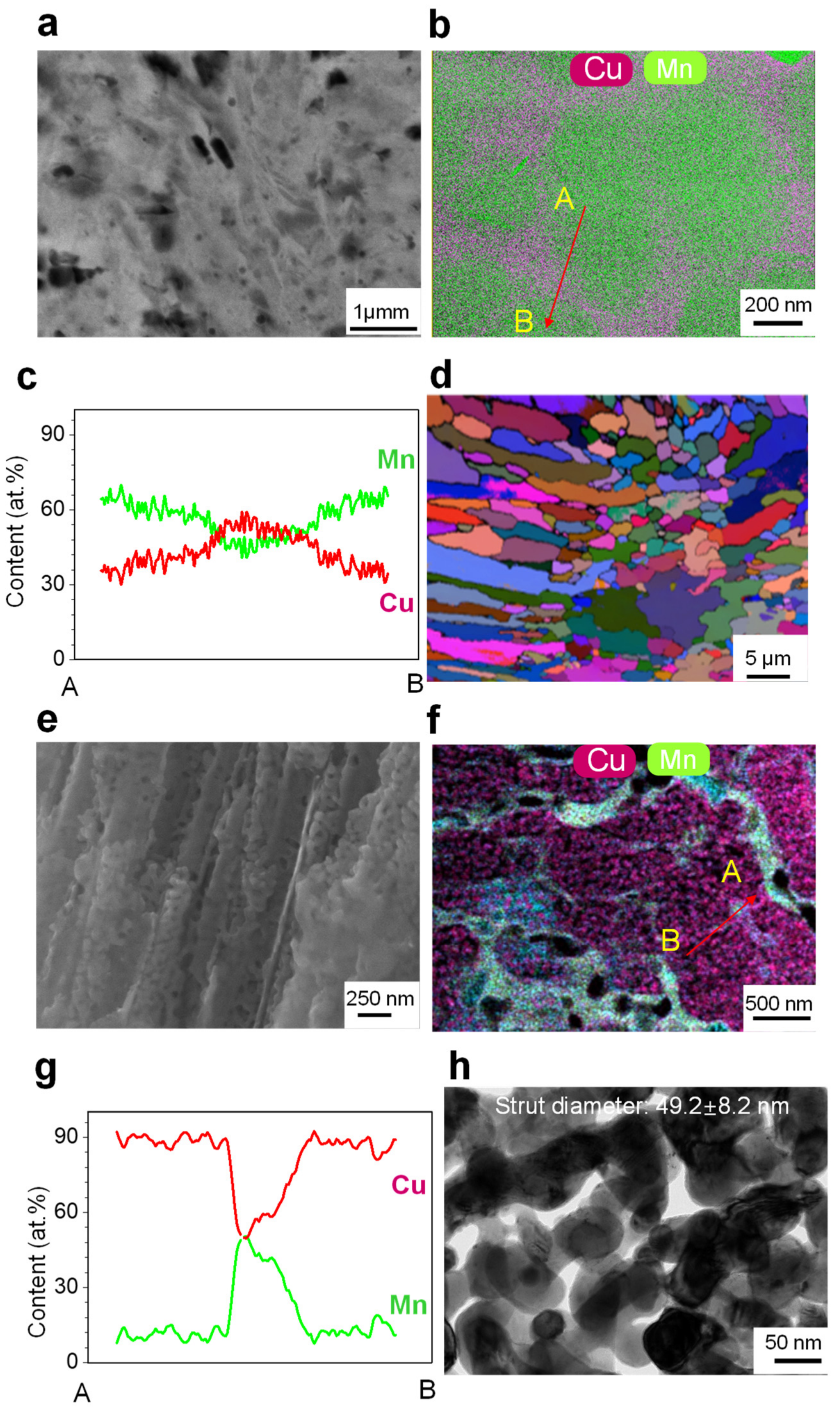

Fig. S4 Characterization of dealloyed topologies and the thermal treated precursor. (a-d) Microstructures and compositions of thermal treated (450°C/30min) precursors of Mn-42.0at.%Cu. (e-h) Dealloyed topologies and corresponding compositions.

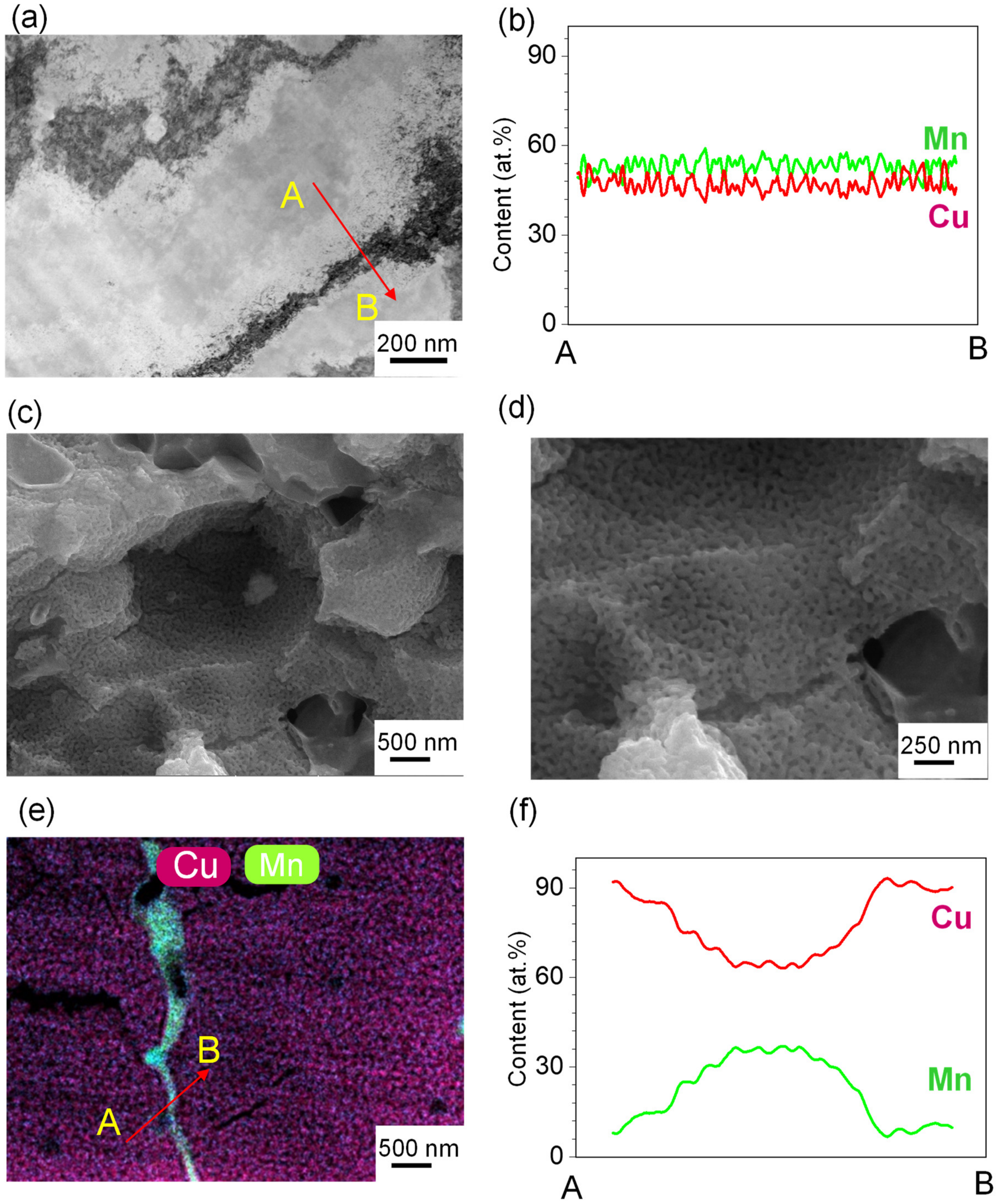

Fig. S5 Characterization of dealloyed topologies and the thermal treated precursor. (a-b) Microstructures and compositions of thermal treated (650°C/30min) precursors of Mn-42.0at.%Cu. (c-d) Dealloyed topologies and corresponding compositions.

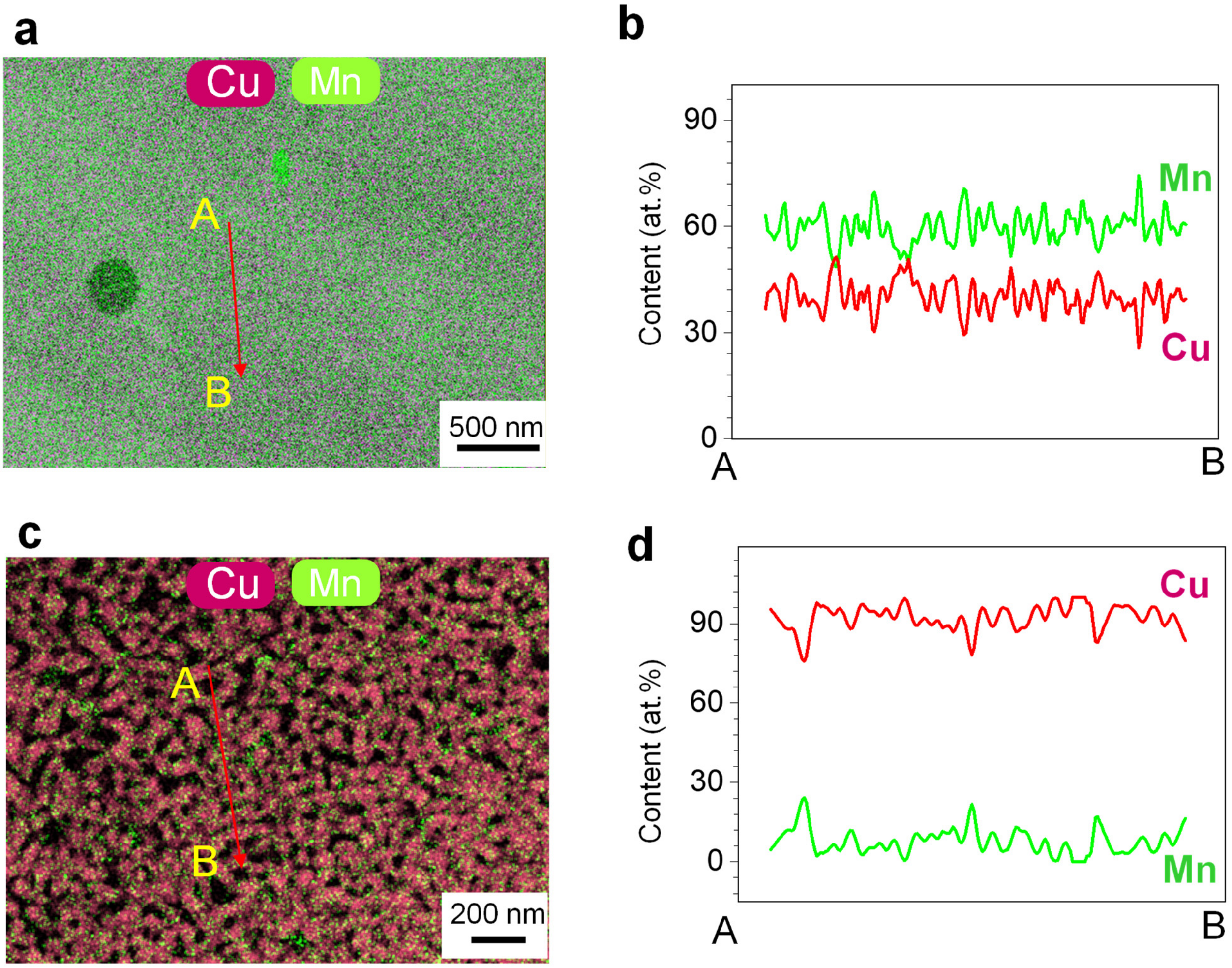

Fig. S6 Characterization of dealloyed topologies and the thermal treated precursor. (a-b) Microstructures and compositions of thermal treated (850°C/30min) precursors of Mn-42.0at.%Cu. (c-d) Dealloyed topologies and corresponding compositions.

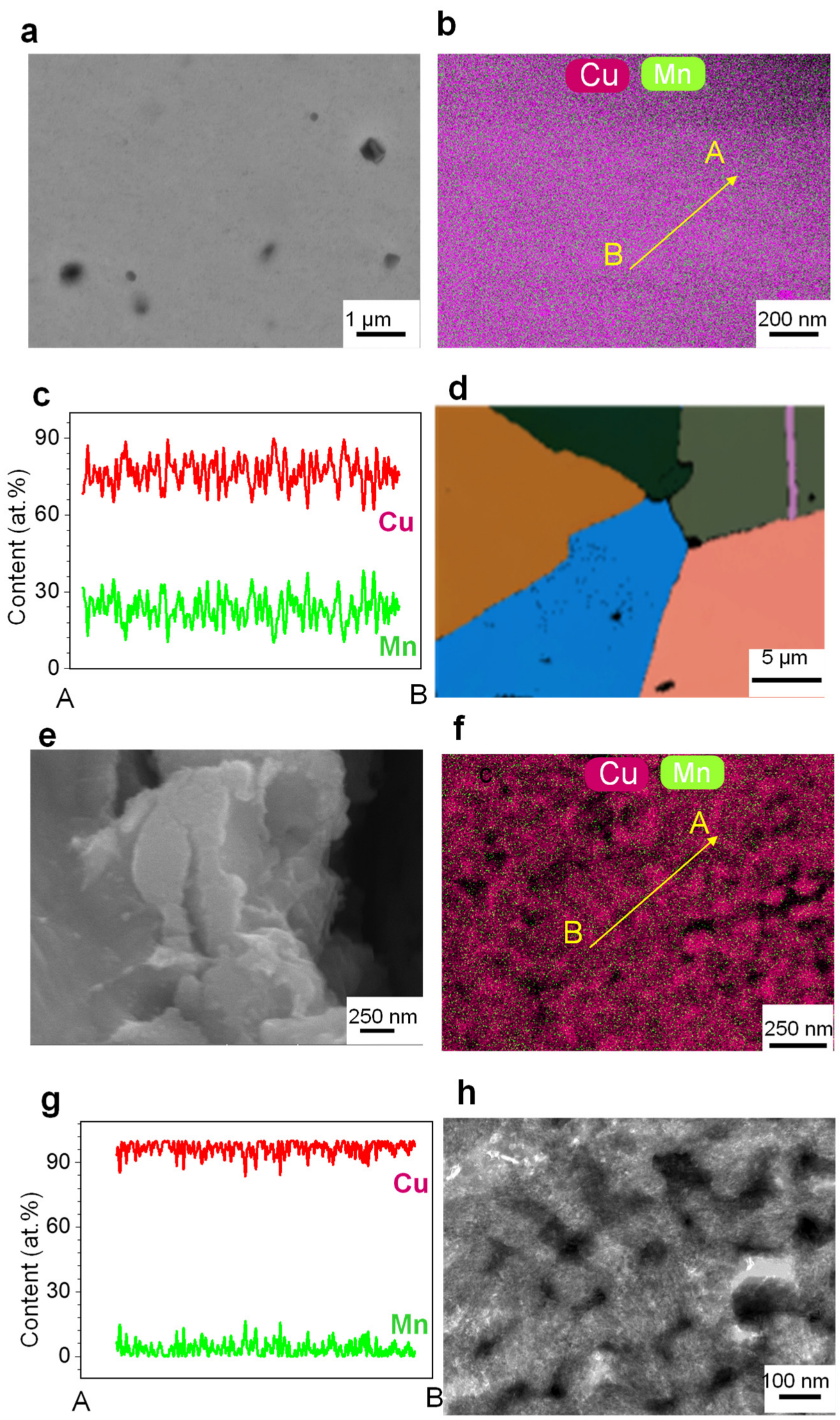

Fig. S7 Characterization of dealloyed topologies and the thermally treated precursor. (a-d) Microstructures and compositions of thermal treated (850℃/72h) precursors of Mn-42.0at.%Cu. (e-h) Dealloyed topologies and corresponding compositions.

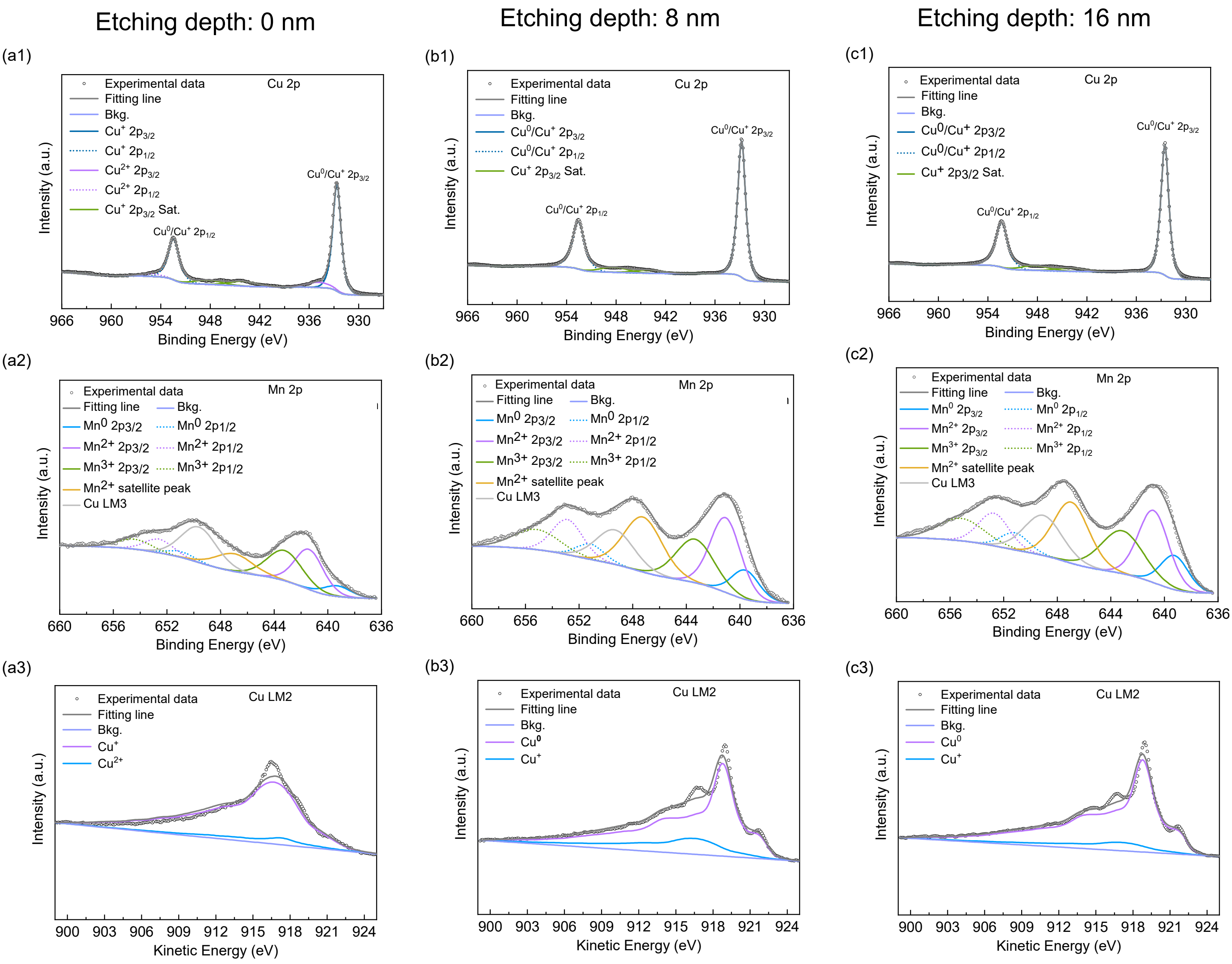

Fig. S8 **XPS analysis of skeletal NPCu to determine the valence states of Cu and Mn.** Panels (a)–(c) represent data collected at sputtering depths of 0 nm, 8 nm, and 16 nm, respectively, with each column corresponding to one depth, while each row displays a specific spectral region: the first row shows Cu 2p, the second row shows Mn 2p, and the third row shows Cu LM2 Auger spectra.

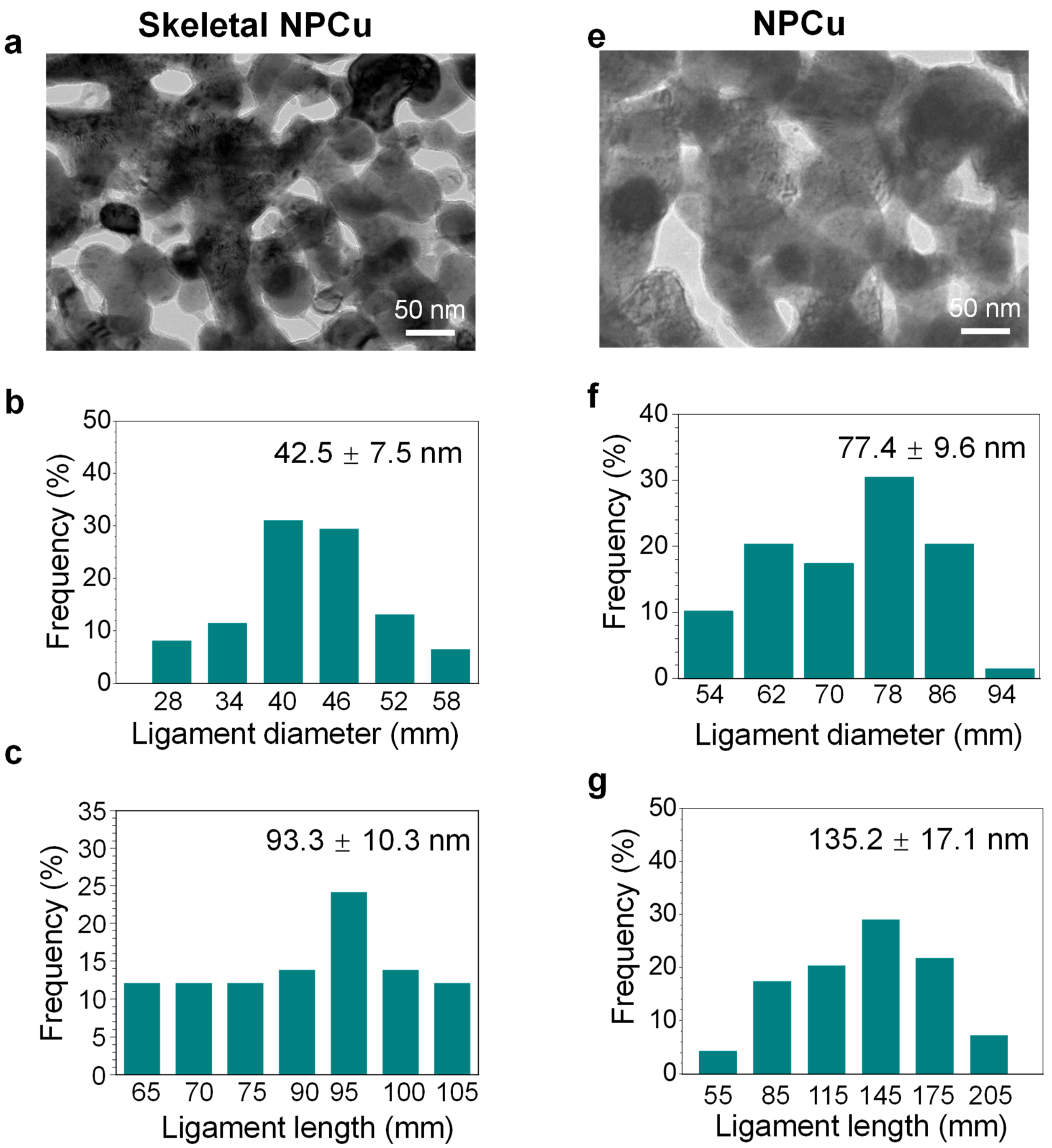

Fig. S9 Statistical analysis of ligament size for skeletal NPCu and NPCu. (a, e) Ligament morphology. (b, f) Ligament diameter. (c, g) Ligamater length. *The data is based on statistical analysis of at least 60 ligaments from multiple TEM-characterized images.*

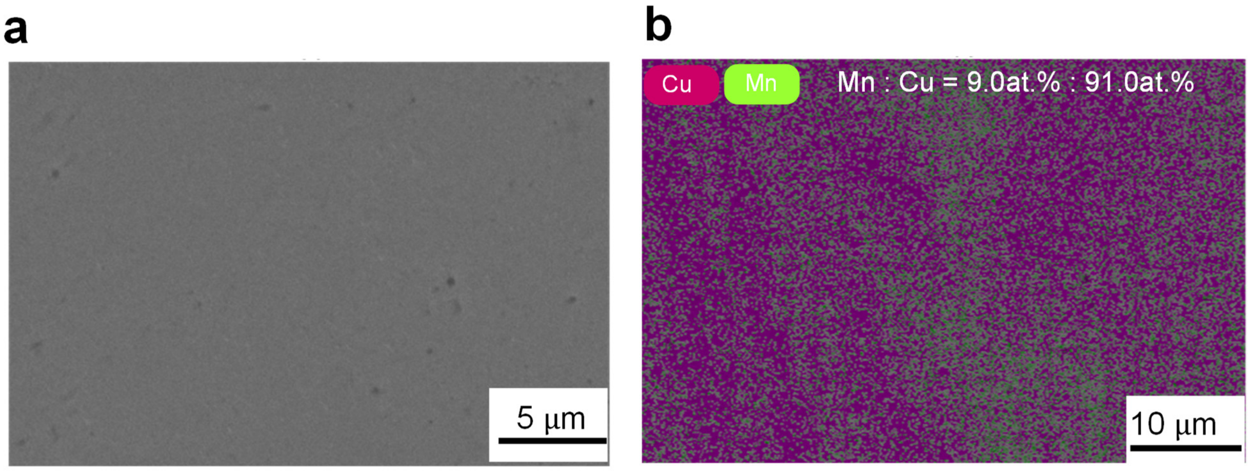

Fig. S10 Characterization of PBF-LB Mn-91.0at.%Cu. (a) Microstructures. (B) EDS map of composition.

4. Mechanical properties and densities of precursors and dealloyed topologies

Fig. S11a displays the pillar-compressed skeletal NPCu strength, measured at 222.4 MPa, which closely aligns with the 200.4 MPa obtained from macro-compression testing in the main text. **Fig. S11**b and Table S2 show that the yield strength of the Mn-42.0at.%Cu precursor is measured at 296.1 MPa, which can be approximated as the inherent strength of the skeleton. **Fig. S11**c and **Table S2** present the strength of the Mn-7.9Cu solid, which corresponds to the final composition of skeletal NPCu. The alloy was printed, and mechanically tested to investigate the relative strength of the skeletal NPCu strut lattice.

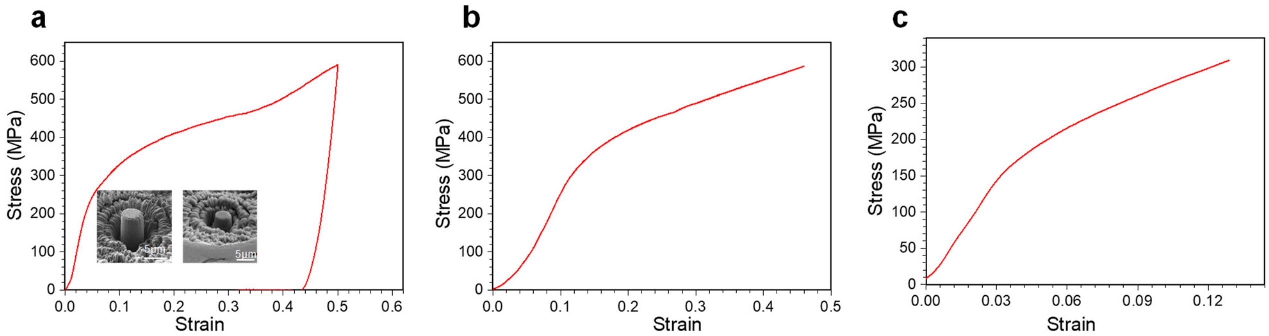

Fig. S11 Compressive curves. (a) Pillar test of skeletal NPCu. (b) Mn-42.0at.%Cu precurosr solid. (c) Mn-91.0at.%Cu solid.

Table S2 The density and yield strength of Mn-91.0at.%Cu and Mn-42.0at.%Cu solid materials.

Solid material	Density (g/cm^3)	Yield strength (MPa)
Mn-91.0at.%Cu	8.7	163
Mn-42.0at.%Cu	6.2	296.1

The relative density calculation of dealloyed topology, including skeletal NPCu and NPCu, denoted as $\rho_{\mathrm{RD\_dealloying}}$, varies across the literature and can be categorized based on two definitions:

a. Mass Ratio Definition

$\rho_{\mathrm{RD\_dealloying}}$ is determined as the ratio of the dealloyed mass ($m_{\mathrm{dealloying}}$) to the precursor mass ($m_{\mathrm{precursor}}$) or or equivalently, the ratio of the dealloyed topology density ($\rho_{\mathrm{dealloying}}$) to the precursor density ($\rho_{\mathrm{precursor}}$) [9], expressed as

$$\rho_{\mathrm{RD\_dealloying}} = \frac{m_{\mathrm{dealloying}}}{m_{\mathrm{precursor}}} = \frac{\rho_{\mathrm{dealloying}}}{\rho_{\mathrm{precursor}}} \qquad \textbf{S7}$$

For a Mn-42.0 at.% Cu precursor (Mn-46 wt.% Cu), this yields a relative density of:

$$\frac{\mathrm{g/cm^3}}{6.2\ \mathrm{g/cm^3}} = 0.54 \qquad \textbf{S8}$$

b. Relative Density Comparison Definition

$\rho_{\mathrm{RD\_dealloying}}$ is defined as the ratio of the nanoporous metal (NPM) relative density ($\rho_{\mathrm{dealloying}}$) to the relative density of a solid material with the same composition ($\rho_{\mathrm{solid\ (equal\ composition)}}$, which differs from $\rho_{\mathrm{precursor}}$ due to compositional changes [12], given by:

$$\rho_{\mathrm{RD\_dealloying}} = \frac{\rho_{\mathrm{dealloying}}}{\rho_{\mathrm{solid\ (equal\ composition)}}} \qquad \textbf{S9}$$

Skeletal NPCu has a composition of Mn-91.0 at.% Cu (Mn-92.1 wt.% Cu), with a solid material density of 8.7 g/cm^3, resulting in a relative density of

For skeletal NPCu with a composition of Mn–91.0 at.% Cu (Mn–92.1 wt.% Cu), the solid material density is 8.7 g/cm^3, resulting in a relative density of:

$$\frac{3.3\ g/cm^3}{8.7 g/cm^3} = 0.38 \qquad \textbf{S10}$$

In this study, we adopt the first definition for relative density calculations, which is based on the mass ratio of the dealloyed material to its precursor. This approach represents a more conservative estimation, as it does not account for the densification effect observed in the skeletal NPCu structure. Consequently, the relative density value for skeletal NPCu in **Table S3** is reported as 0.54 rather than 0.38, ensuring that our calculations reflect the least favorable yet most rigorous assessment of material porosity.

Table S3 **Densities and strengths of skeletal NPCu and NPCu.**

Dealloyed topology	Density (g/cm^3) $\rho_{\text{RD\_dealloying}}$	Relative density	Yield strength (MPa)	Maximum strength
Skeletal NPCu	3.3	0.54	200.4 ± 15.2	239.3± 21.2
NPCu	2.9	0.46	107.5± 8.9	138 ± 10.5

The skeletal NPCu strut lattice can be considered a form of hierarchical lattice, where the skeletal NPCu exists at a smaller scale, while the overall lattice structure is at a larger scale. For a general hierarchical lattice, the relative density is typically calculated using the product formula [26]:

$$\rho_{RD} = \rho_{\text{RD, I}} \times \rho_{\text{RD, II}} \qquad \textbf{S11}$$

where $\rho_{\text{RD, I}}$ and $\rho_{\text{RD, II}}$ represent the relative densities at the larger and smaller scales, respectively.

However, the density calculation for skeletal NPCu remains a subject of debate, as discussed earlier. In this study, the primary objective of calculating relative density is to ensure that the skeletal NPCu strut lattice and the comparator lattice are evaluated under identical density conditions, allowing for a justified performance comparison. Based on this requirement, we adopt the relative density definition:

$$\rho_{RD} = \rho_{\text{Hierarchical lattice}}/\rho_{\text{Precursor}} \qquad \textbf{S12}$$

to maintain consistency in our comparative analysis. Therefore, the values in the last column of **Table S4** for hierarchical lattices are 0.316.

Table S4 **Relative densities at each scale of skeletal NPCu strut lattices and the comparator lattices.**

Lattices		Density (g/cm^3)	I-scale (Larger-scale) Topology	$\rho_{\text{RD, I}}$	II-scale (Smaller-scale) Topology	$\rho_{\text{RD, II}}$	ρ_{RD}
Skeletal NPCu strut lattices	square honeycomb	2.75	Square honeycomb	0.80	Skeletal NPCu	≈ 54%	0.316
	cubic	2.75	Cubic	0.81			0.316
	gyroid	2.65	Gyroid	0.80			0.305
Comparator lattices	square honeycomb	2.72	Square honeycomb	0.313	N/A		0.313
	cubic	2.75	Cubic	0.316			0.316
	square gyroid	2.77	Gyroid	0.318			0.318

5. Strengthening mechanism

The skeleton and the surrounding nanoporous copper (NPCu) phases work together as a composite material, with their respective volume fractions and strengths determining the overall strength of skeletal NPCu. The total strength of skeletal NPCu can be approximately estimated using the rule of mixtures. Here, V_{Skeleton} and V_{NPCu} represent the volume fractions of the skeleton and NPCu, respectively, and the intrinsic strengths of the materials, σ_{Skeleton} and σ_{NPCu} represent their inherent strength. As indicated by the atomic force microscopy (AFM) characterization in **Fig. S12**, these inherent strengths vary significantly, which influences the overall strength of the composite.

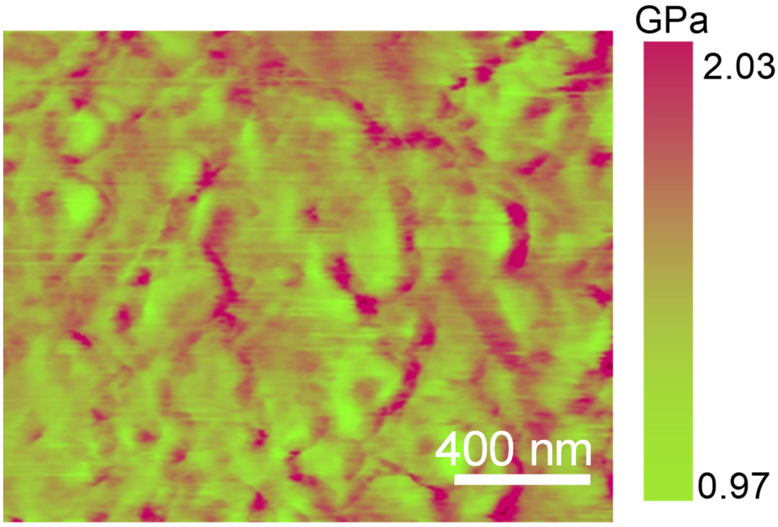

Fig. S12 The mechanical properties of skeleton and skeleton-surrounded NPCu by atom force microscope.

As shown earlier in Fig. 3b, the Cu content in the skeleton region, NPCu region, and global region is 49.5 at.% and 99.1 at.%, respectively. The volume fractions of the skeleton (V_{Skeleton}) and NPCu (V_{NPCu}) satisfy the relation $V_{\text{Skeleton}} + V_{\text{NPCu}} = 1$. Using ***Eq. S13***, yielding 16.3 wt.% and 83.7 wt.%, respectively.

$$V_{\text{Skeleton}} \times 49.5\text{at.}\% + V_{\text{NPCu}} \times 99.1\text{at.}\% = 91.0\text{at.}\% \qquad \text{S13}$$

The pre-embedded structural constraints observed in **Fig. 3** manifest as dual strengthening pathways: (i) direct load-bearing by the Mn-42 at.%Cu skeleton and (ii) indirect ligament refinement through dealloying confinement. These mechanisms synergistically follow the rule of mixtures neglecting interfacial synergy effects: direct reinforcement by the continuous skeleton and indirect ligament refinement strengthening, forming a composite-like architecture [9]. Analogous to the bamboo framework in Tulou walls, the reinforcing skeleton not only provides intrinsic load-bearing capacity but also governs the nanostructural evolution of the nanoporous phase, establishing a bimodal

strengthening system. These phases contribute to the overall strength based on their respective volume fractions and intrinsic strengths, as illustrated in **Fig. S12**. The total strength follows the rule of mixtures, as formulated in ***Eq. S14***,where the first and second items represent the direct and indirect contribution, respectively.

$$\sigma_{\text{skeletal NPCu}} = \sigma_{\text{Skeleton}} \times V_{\text{Skeleton}} + \sigma_{\text{NPCu}} \times V_{\text{NPCu}} \quad \textbf{S14}$$

- **Direct skeleton strengthening**

The Mn-42 at.%Cu skeleton exhibits an inherent yield strength of 296.1 MPa (equivalent to its precursor alloy, **Fig. S11*b***). With a volume fraction of 16.3% (V_{Skeleton}), its direct strengthening contribution is calculated as:

$$\sigma_{\text{Skeleton}} \times V_{\text{Skeleton}} = 296.1 \times 16.3\% = 48.3\ \text{MPa} \quad \textbf{S15}$$

- **Indirect ligament refinement strengthening**

Critically, the skeleton retains residual Cu that restricts nanoporous coarsening during dealloying. This refines the NPCu ligament diameter from 77.4 nm (skeleton-free NPCu, **Fig. S9*d-f***) to 42.5 nm (skeleton-constrained case, **Fig. S9*a-c***). The NPCu phase strength (σ_{NPCu}) is governed by a ligament size effect and cellular topology. They are determined by Hall-Petch equation [7, 27] and bending-dominated Gibson-Ashby model [28], determined by expressed through:

$$\sigma_{\text{Ligament}} = (\sigma_0 + kd^{-0.5}) \quad \textbf{S16}$$

$$\sigma_{\text{NPCu}} = \sigma_{\text{Ligament}}(\rho_{\text{RD\_dealloying}})^2 \quad \textbf{S17}$$

where σ_0 is the intrinsic strength of material, k is the material constant. For copper, σ_0 =16 MPa, k = 0.25 MPa/m [29], d=42.5 nm, and $\rho_{\text{RD\_dealloying}} = 0.54$, the indirect contribution $\sigma_{\text{NPCu}} \times V_{\text{NPCu}}$ reaches 136.0 MPa. When substituting d=77.4 nm, which is the ligament diameter for skeletal-free NPCu, the calculated value reduces to 101.2 MPa. It indicates the 34.8 MPa (136.0 -101.2 = 34.8 MPa) higher than skeleton-free NPCu due to ligament refinement.

- **Synergistic strengthening validation**

The combined contributions (48.3 MPa + 136.0 MPa = 184.3 MPa) align closely with experimental measurements (200.4 MPa). Remarkably, the skeleton contributes 45% of total strength through:

1. Direct load transfer (48.3 MPa)

2. Ligament refinement (34.8 MPa via dd reduction)

The nanoscale skeleton acts as a shear band deflector (**Fig. S12**), constraining plastic deformation through interfacial stress redistribution. This Tulou-inspired architecture demonstrates that geometric confinement and nanostructural control synergistically enhance mechanical resilience beyond conventional NPCu.

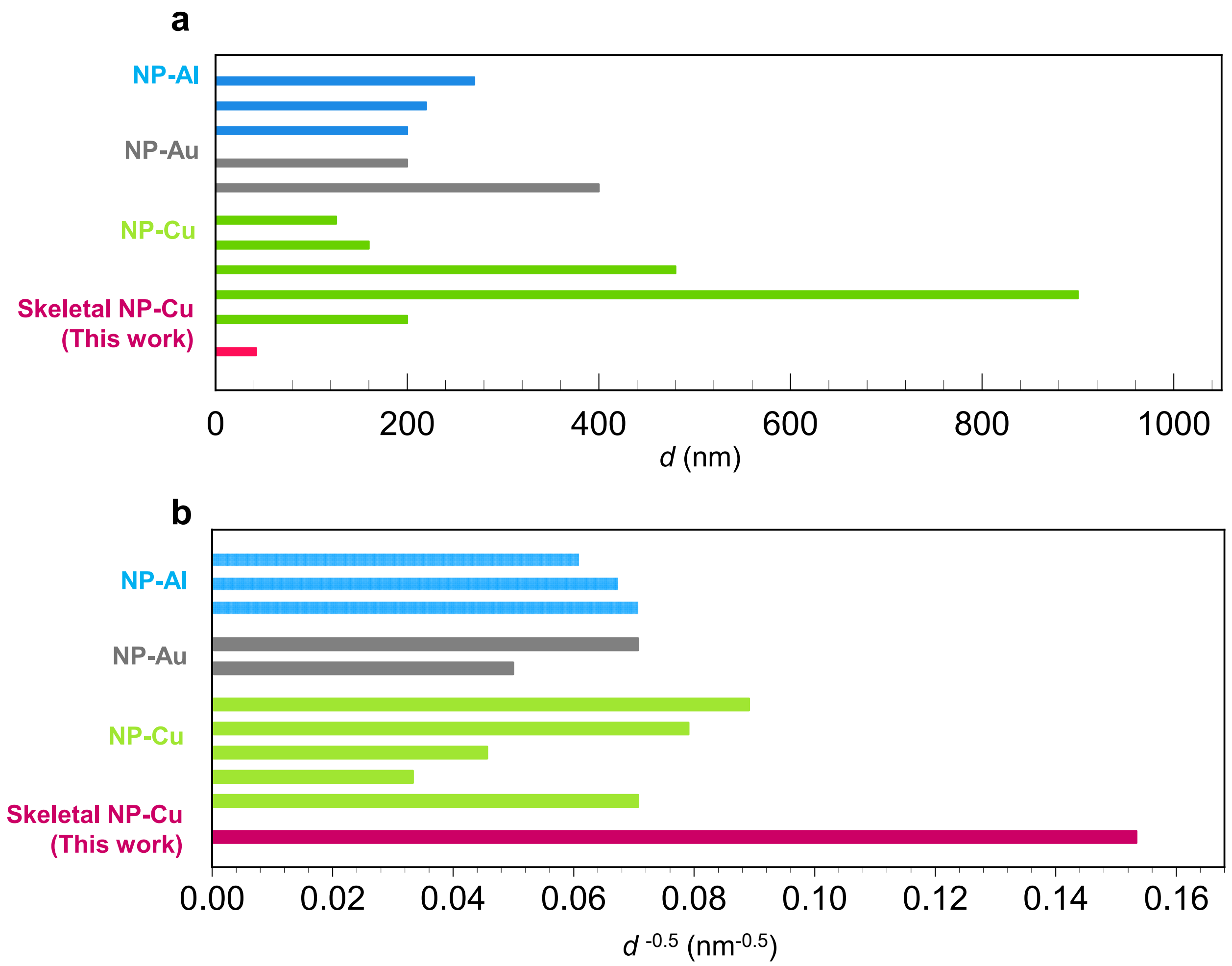

Fig. S13 Comparison of ligament diameters of skeletal NPCu and reported nanoporous metals. (a) Direct comparison of average ligament diameter (d) values. (b) Comparison of $d^{-0.5}$ values, highlighting the inverse-square-root trend relevant to surface-area-dependent scaling. Literature data: [3, 9, 30-32]

The presence of the skeleton plays a crucial role in shortening and deflecting shear bands, thereby slowing their propagation. This mechanism is similar to grain boundary strengthening in metallurgy [33]. However, this effect is difficult to observe directly during the compression deformation of nanoscale skeletal NPCu. To better visualize the mechanism, we designed and 3D-printed porous polymer structures with skeletons (**Fig. S14a-b**), mimicking the topology of skeletal NPCu while

excluding nanoscale effects. This topology maintained structural integrity (see inset of **Fig. S14c**) beyond the yield point and exhibited strain hardening (see the compressive curve in **Fig. S14c**). For comparison, we also designed and fabricated porous polymers without skeletons, maintaining the same relative densities (**Fig. S14d-e**). These structures demonstrated lower strength (7.2 MPa vs. 5.1 MPa) and poor structural integrity (see inset of **Fig. S14f**).

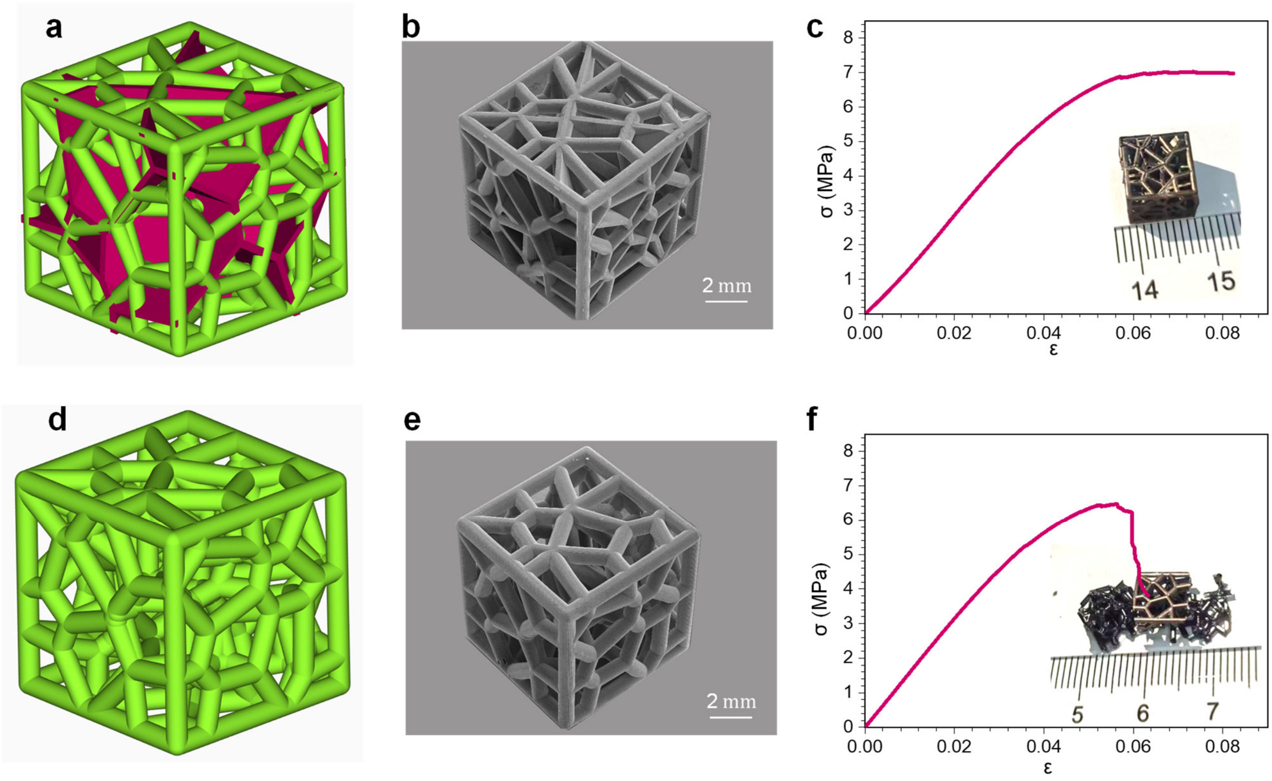

Fig. S14 Evaluation of the skeleton's influence on crack propagation using 3D-printed porous polymer topologies. (a-c) Porous structure with skeleton topology mimicking skeletal NPCu, and the corresponding compressive curve. (d-f) Porous structure without skeleton topology for comparison, and the corresponding compressive curve. The relative densities of both (a) and (d) are set at 35%.

6. Precursor design and printing

Table S5 The topological parameters of skeletal NPCu and comparator lattices.

Lattices		Strut thickness (mm)	Unit size (mm)	Arrays	Three-dimensional size	CAD relative density	Constituent material
Skeletal NPCu Strut lattices	Square honeycomb	0.58	1.2	4 × 4	4.2 × 4.2 × 9 mm^3	80.0%	Mn-42.0at.%Cu
	cubic	0.78	1.2	4 × 4 × 9	4.3 × 4.3 × 10.3 mm^3	81.0%	Mn-42.0at.%Cu
	gyroid	/	4	2 × 2 × 3	8 × 8 × 12 mm^3	80.0%	Mn-42.0at.%Cu
Comparator lattices	Square honeycomb	0.16	1.2	4 × 4	3.75 × 3.75 × 9 mm^3	29.4%	Mn-91.0at.%Cu
	cubic	0.37	1.2	4 × 4 × 9	3.97 × 3.97 × 9.97 mm^3	29.5%	Mn-91.0at.%Cu
	gyroid		4	2 × 2 × 3	8 × 8 × 12 mm^3	28.8%	Mn-91.0at.%Cu

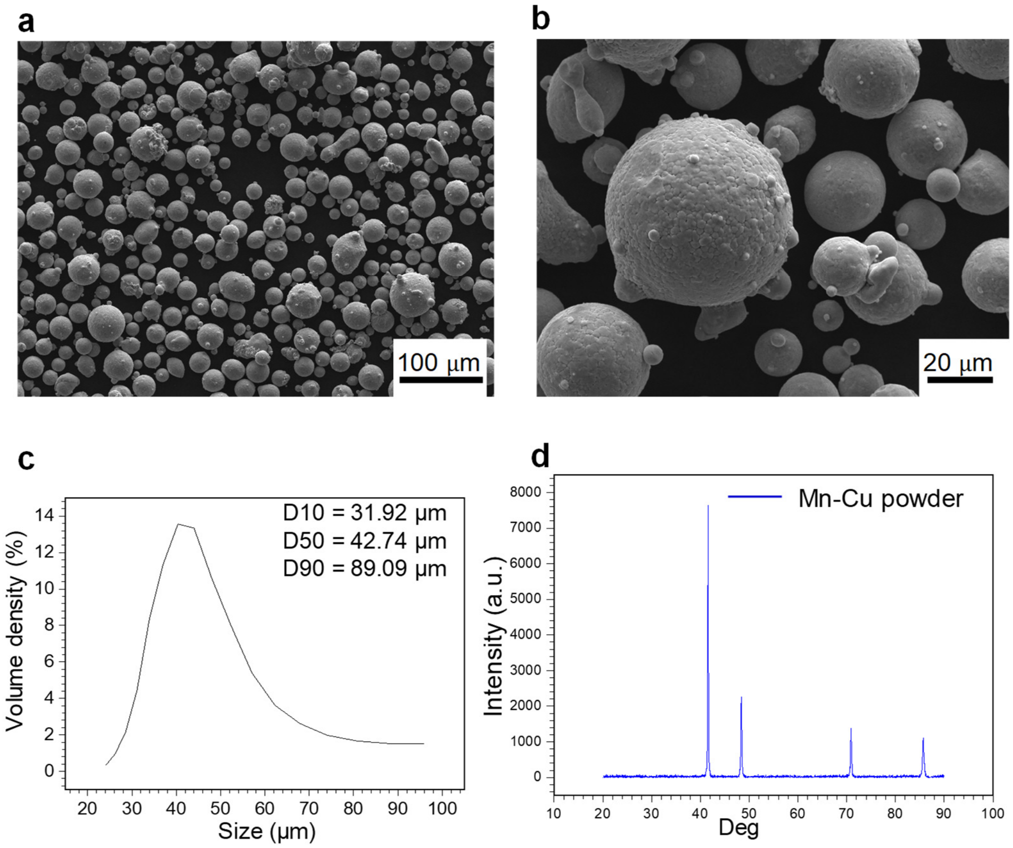

Fig. S15 Characterization of Mn-42.0at.%Cu powder. (a-b) The powder morphology. (c) The powder size distribution. (d) XRD showing phases of Mn–42.0 at.% Cu powder.

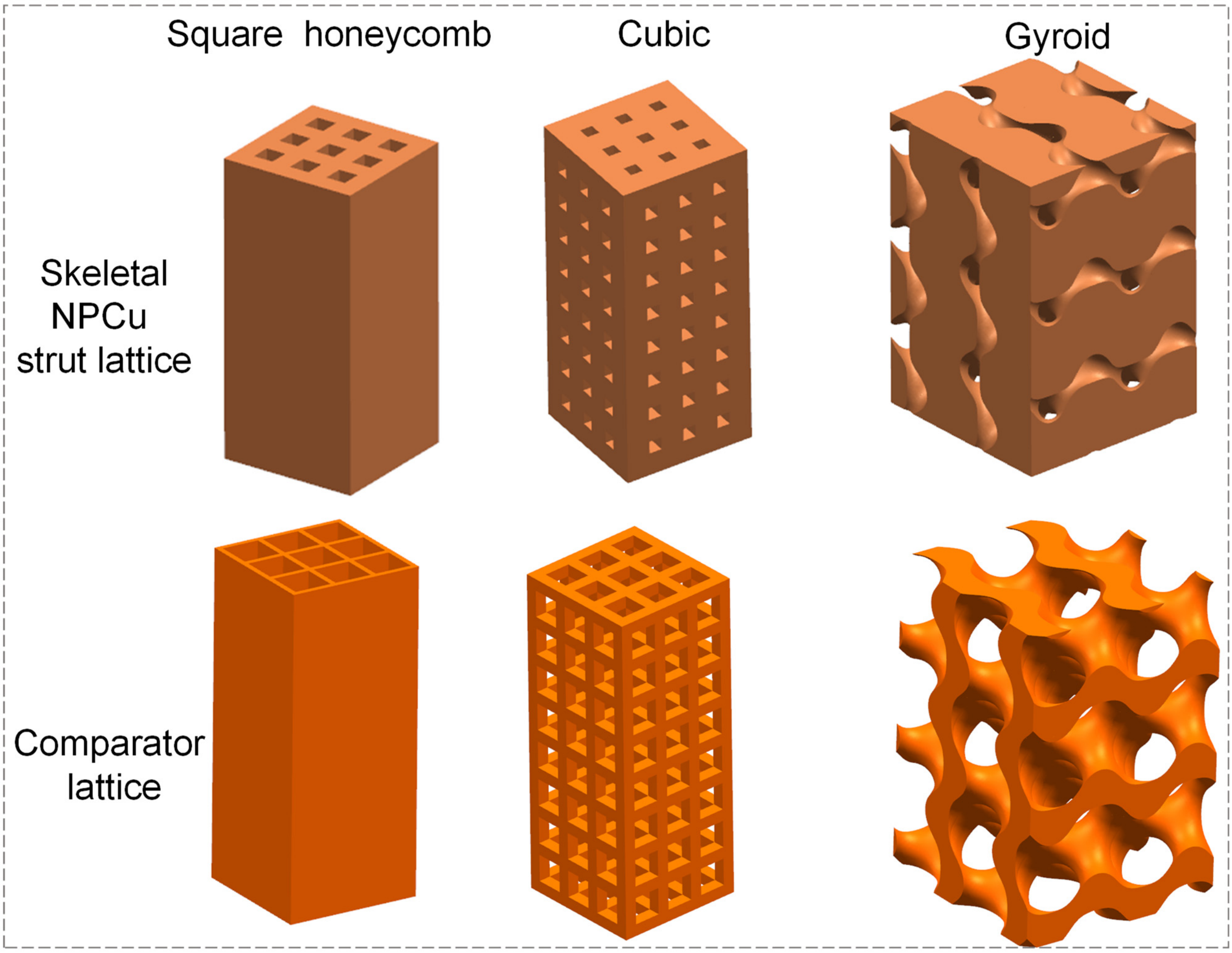

Fig. S16 **CAD models of skeletal NPCu strut (top row) and comparator (bottom row) lattices**

Table S6 **Compositions of printed Mn-Cu alloys.**

Designed (nominate) composition (wt.%)	Manufacturing method	Measured composition after printing
Cu : Mn=91at.% : 9at.%	Mixing	Cu : Mn=91.0at.% : 9.0at.%
Cu : Mn=40.0at.% : 60.0at.%	Argon gas atomizing	Cu : Mn=42.4at.% : 57.6at.%

Table S7 **Printing parameters of Mn-Cu lattices.**

Laser beam diameter, *d* (mm)	Power, *P* (W)	Scanning speed, *v* (mm/s)	Hatch spacing, *d* (μm)	Layer thickness, *t* (μm)
55 μm	90	700	105	25

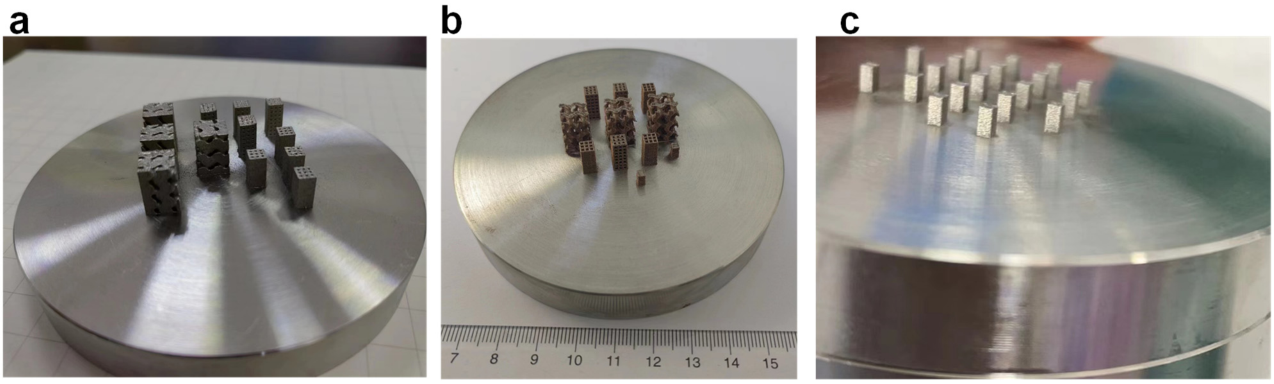

Fig. S17 Printed samples. (a) The Mn-42 at.%Cu lattices, which are transformed into skeletal NPCu strut lattice through post dealloying. (b) The Cu-9.0 at.% Mn comparator lattice. (c) The Mn-42 at.%Cu solid precursor sample.

Refereces

[1] Y. Ji, Y. Xing, F. Zhou, X. Li, Y. Chen, L.-H. Shao, The Mechanical Characteristics of Monolithic Nanoporous Copper and Its Composites, Advanced Engineering Materials 20(10) (2018).

[2] F. Chen, X. Chen, L. Zou, Y. Yao, Y. Lin, Q. Shen, E.J. Lavernia, L. Zhang, Fabrication and mechanical behavior of bulk nanoporous Cu via chemical de-alloying of Cu–Al alloys, Materials Science and Engineering: A 660 (2016) 241-250.

[3] L. Wang, H. Xie, J.-C. Shao, H. Yang, P.-Z. Feng, H.-J. Jin, High-strength hierarchical-structured bulk nanoporous Cu prepared by dealloying and spark plasma sintering, Scripta Materialia 203 (2021) 114114.

[4] F. Chen, Fabrication of porous metal copper by dealloying and study of porous structure via synchrotron radiation, 2020.

[5] J.L. Smith, Bactericidal copper nanostructures by chemical dealloying, 2020.

[6] I.C. Cheng, A.M. Hodge, Strength scale behavior of nanoporous Ag, Pd and Cu foams, Scripta Materialia 69(4) (2013) 295-298.

[7] S. Shi, Y. Li, B.N. Ngo-Dinh, J. Markmann, J. Weissmüller, Scaling behavior of stiffness and strength of hierarchical network nanomaterials, Science 371(6533) (2021) 1026-1033.

[8] C. Wang, S. Zhu, Y. Liang, Z. Cui, S. Wu, C. Qin, S. Luo, A. Inoue, Understanding the macroscopical flexibility/fragility of nanoporous Ag: Depending on network connectivity and micro-defects, Journal of Materials Science & Technology 53 (2020) 91-101.

[9] W. Yang, Z.P. Luo, W.K. Bao, H. Xie, H.J. Jin, Light, strong, and stable nanoporous aluminum with native oxide shell, Science Advances 7(28) (2021) eabb9471.

[10] C.A. Volkert, E.T. Lilleodden, D. Kramer, J. Weissmüller, Approaching the theoretical strength in nanoporous Au, Applied Physics Letters 89(6) (2006).

[11] J. Biener, A.M. Hodge, J.R. Hayes, C.A. Volkert, L.A. Zepeda-Ruiz, A.V. Hamza, F.F. Abraham, Size effects on the mechanical behavior of nanoporous Au, Nano letters 6(10) (2006) 2379-2382.

[12] H. Kashani, M. Chen, Flaw-free nanoporous Ni for tensile properties, Acta Materialia 166 (2019) 402-412.

[13] H. Liu, Y. Liu, S. Lu, Y. Zhang, H. Chen, Y. Chen, M. Qian, Alloy solidification: assessment and improvement of an easy-to-apply model, Journal of Materials Science & Technology 130 (2022) 1-11.

[14] W. Kurz, M. Rappaz, R. Trivedi, Progress in modelling solidification microstructures in metals and alloys. Part II: dendrites from 2001 to 2018, International Materials Reviews 66(1) (2021) 30-76.

[15] T. Gong, Y. Chen, S. Li, Y. Cao, D. Li, X.-Q. Chen, G. Reinhart, H. Nguyen-Thi, Revisiting dynamics and models of microsegregation during polycrystalline solidification of binary alloy, Journal of Materials Science & Technology 74 (2021) 155-167.
[16] Y. Iijima, K.-i. Hirano, K. Sato, Interdiffusion in Cu–Mn Alloys, Transactions of the Japan Institute of Metals 18(12) (1977) 835-842.
[17] C. Wei, Y. Liu, Y. Han, J. Wan, K. Yang, Microstructures of eutectic Sn–Ag–Zn solder solidified with different cooling rates, Journal of alloys and compounds 464(1-2) (2008) 301-305.
[18] K. Eckler, R. Cochrane, D. Herlach, B. Feuerbacher, M. Jurisch, Evidence for a transition from diffusion-controlled to thermally controlled solidification in metallic alloys, Physical Review B 45(9) (1992) 5019.
[19] J. Kittl, P. Sanders, M. Aziz, D. Brunco, M. Thompson, Complete experimental test of kinetic models for rapid alloy solidification, Acta materialia 48(20) (2000) 4797-4811.
[20] H. Hyer, L. Zhou, A. Mehta, Y. Sohn, Effects of alloy composition and solid-state diffusion kinetics on powder bed fusion cracking susceptibility, Journal of Phase Equilibria and Diffusion 42 (2021) 5-13.
[21] C. Wang, C. Beckermann, A unified solute diffusion model for columnar and equiaxed dendritic alloy solidification, Materials Science and Engineering: A 171(1-2) (1993) 199-211.
[22] A. Roy, R. Chhabra, Prediction of solute diffusion coefficients in liquid metals, Metallurgical Transactions A 19 (1988) 273-279.
[23] W. Pan, R. Popescu, H. Meyerheim, D. Sander, O. Robach, S. Ferrer, M.-T. Lin, J. Kirschner, Stress and structure of c (2× 2) and p 2 gg (4× 2) Mn⁄Cu (001) surface alloys, Physical Review B—Condensed Matter and Materials Physics 71(17) (2005) 174439.
[24] V. Ramanuj, Numerical Modeling of an Alloy Droplet Deposition with Non-Equilibrium Solidification, (2016).
[25] R.M. Sharp, Studies of Solute Distribution at Solid-liquid Interfaces, University of Oxford1971.
[26] M.F. Ashby, The properties of foams and lattices, Phil. Trans. R. Soc. A 364(1838) (2006) 15-30.
[27] L.-Z. Liu, H.-J. Jin, Scaling equation for the elastic modulus of nanoporous gold with “fixed” network connectivity, Applied Physics Letters 110(21) (2017).
[28] M. Ashby, L. Gibson, Cellular solids: structure and properties, Press Syndicate of the University of Cambridge, Cambridge, UK, 1997.
[29] S. Lefebvre, B. Devincre, T. Hoc, Simulation of the Hall–Petch effect in ultra-fine grained copper, Materials Science and Engineering: A 400-401 (2005) 150-153.
[30] P. Zhu, Z. Wu, Y. Zhao, Hierarchical porous Cu with high surface area and fluid permeability, Scripta Materialia 172 (2019) 119-124.
[31] A.M. Hodge, J. Biener, J.R. Hayes, P.M. Bythrow, C.A. Volkert, A.V. Hamza, Scaling equation for yield strength of nanoporous open-cell foams, Acta Materialia 55(4) (2007) 1343-1349.
[32] M. Hakamada, M. Mabuchi, Mechanical strength of nanoporous gold fabricated by dealloying, Scripta Materialia 56(11) (2007) 1003-1006.
[33] T. Zhu, J. Li, Ultra-strength materials, Progress in Materials Science 55(7) (2010) 710-757.
